# Lymphovascular invasion and histologic grade are associated with specific genomic profiles in invasive carcinomas of the breast

**DOI:** 10.1007/s13277-014-2786-z

**Published:** 2014-11-13

**Authors:** Felipe Fidalgo, Tatiane Cristina Rodrigues, Mabel Pinilla, Amanda Gonçalves Silva, Maria do Socorro Maciel, Carla Rosenberg, Victor Piana de Andrade, Dirce Maria Carraro, Ana Cristina Victorino Krepischi

**Affiliations:** 1 International Research Center, AC Camargo Cancer Center, São Paulo, Brazil; 2 Department of Genetics and Evolutionary Biology, Institute of Biosciences, University of São Paulo, São Paulo, Brazil; 3 Department of Breast Cancer, AC Camargo Cancer Center, São Paulo, Brazil; 4 Department of Surgical and Investigative Pathology, AC Camargo Cancer Center, São Paulo, Brazil

**Keywords:** Invasive ductal carcinomas of the breast, Breast cancer, DNA copy number aberrations, CNA, Lymphovascular invasion, Histologic grade III, Gene expression, *S100A8*, *MMP1*, *MED1*, *ADAMTS3*, *HSD17B12*

## Abstract

Lymphovascular invasion (LVI) and histologic grade are clinical parameters of high prognostic value in breast cancer and indicate the level of tumor aggressiveness. Many studies have focused on the association of breast cancer subtypes with gene expression and chromosomal profiles, but considerably less genomic information is available regarding traditional prognostic factors such as histologic grade and LVI. We studied by array-CGH a group of 57 invasive ductal carcinomas of the breast to outline the DNA copy number aberration (CNA) profile linked to high histologic grades and LVI. Selected CNAs were validated using real-time quantitative PCR (qPCR). Furthermore, gene expression analysis was performed in a subset of 32 of these tumors, and findings were integrated with array-CGH data. Our findings indicated an accumulation of genomic alterations in high-grade breast tumors compared to low-grade samples. Grade III tumors showed higher number of CNAs and larger aberrations than low-grade tumors and displayed a wide range of chromosomal aberrations, which were mainly 5p, 8q, 10p, 17q12, and 19 gains, and 3p, 4, 5q proximal, 9p, 11p, 18q, and 21 losses. The presence of LVI, a well-established prognostic marker, was not significantly associated with increased genomic instability in comparison to breast tumors negative for LVI, considering the total number of chromosomal alterations. However, a slightly increase in the frequency of specific alterations could be detected in LVI-positive group, such as gains at 5p, 16p, 17q12, and 19, and losses at 8p, 11q, 18q, and 21. Three newly reported small-scale rearrangements were detected in high-risk tumors (LVI-positive grade III) harboring putative breast cancer genes (amplicons at 4q13.3 and 11p11.2, and a deletion at 12p12.3). Furthermore, gene expression analysis uncovered networks highlighting *S100A8*, *MMP1*, and *MED1* as promising candidate genes involved in high-grade and LVI-positive tumors. In summary, a group of genomic regions could be associated with high-risk tumors, and expression analysis pinpointed candidate genes deserving further investigation. The data has shed some light on the molecular players involved in two highly relevant prognostic factors and may further add to the understanding of the mechanisms of breast cancer aggressiveness.

**Electronic supplementary material:**

The online version of this article (doi:10.1007/s13277-014-2786-z) contains supplementary material, which is available to authorized users.

## Introduction

Breast cancer is a significant cause of cancer-related death worldwide [1]. Despite significant advances in diagnosis and treatment, several considerable clinical and scientific problems remain unresolved. Invasive ductal carcinomas (IDC) of no special type (NST) represent 80 % of all invasive tumors of the breast. IDC can be associated with a poorer prognosis than certain special types of breast cancer (such as tubular and mucinous carcinomas) and shows significant biological heterogeneity. Traditional variables, such as tumor size, axillary nodal status, histologic grade, and lymphovascular invasion (LVI) status, are part of risk assessment and deliver significant prognostic information, albeit with limited predictive value [2–6].

The histologic grade [Scarff-Bloom-Richardson (SBR)] modified by Elston and Ellis (modified SBR or Nottingham system) is a widely validated prognostic factor recommended by the Union for International Cancer Control (UICC, 2009). This index grades phenotypic aggressiveness according to morphological criteria based on tubular formation, nuclear pleomorphism, and mitotic counting [7]. Although many studies have focused on the association of breast cancer subtypes with gene expression and chromosomal profiles, considerably less genomic information is available regarding traditional prognostic factors such as histologic grade and LVI [8–12].

Recent studies point to the significance of DNA copy number aberrations (CNAs) in the etiology of cancer with the number and complexity of these aberrations being indicative of overall prognosis [10, 13–15]. CNA investigation may assist the identification of regions containing oncogenes and tumor suppressor genes [16]. Grade III breast tumors frequently harbor gains at 3q and 5p, and 8q amplifications [10, 11]. More recently, a 19q12 amplification was detected, primarily associated with grade III breast tumors in estrogen-negative samples, which encompasses the *CCNE1* gene, among others [17]. Grade I tumors show less complex karyotypes with recurrent gain of 16p, as found in estrogen-positive tumors [11, 18]. However, to the best of our knowledge, no study has investigated the genomic profile of CNAs related to the presence of LVI in breast carcinomas.

We performed a study outlining the CNA and gene expression patterns of invasive carcinomas of the breast to identify genomic alterations and differentially expressed genes linked to high histologic grades and LVI. We suggest several genes as potential biomarkers of breast cancer aggressiveness with the ultimate goal of improving patient care.

## Materials and methods

### Study approval and patient samples

This retrospective study was approved by the local Ethics and Research Committee of A.C. Camargo Cancer Center, São Paulo, Brazil (#1448/10), and informed consents were obtained from all patients. Frozen samples from 57 invasive ductal carcinomas of the breast were retrieved from the A.C. Camargo Cancer Center Biobank for DNA and RNA extractions. All selected carcinomas were tested for estrogen receptor, progesterone receptor, and HER2 status, and analyses followed the ASCO/CAP guidelines [19, 20]. Detailed clinical characteristics of the 57 breast carcinomas are given in Supplementary Table S1. These tumors were selected because of their availability as frozen samples.

### DNA and RNA isolation

Genomic DNA was extracted according to the procedure of the Biobank of the institution [21]. Sample quality and quantity were assessed using NanoDrop (Thermo Scientific, Waltham, MA, USA), and molecular weight was checked by electrophoresis in agarose gels. RNA was obtained from epithelial cells from invasive ductal carcinomas samples captured by laser microdissection using the PixCell II LCM system (Arcturus Engineering, Mountain View, CA, USA). Only RNA samples with optical density of approximately 2.0 and RNA integrity number >5.0 were used for microarray experiments [22].

### Comparative genome hybridization based on microarrays

We performed comparative genomic hybridization based on microarrays (array-CGH) in a commercial whole-genome 60K platform containing 60,000 oligonucleotide probes (Agilent Technologies, Santa Clara, CA, USA; design 21924). A commercially available pool of healthy human female DNA (Promega, Madison, WI, USA) was used as the reference DNA. The experimental procedure was performed as recommended by the manufacturer. Scanned images were processed using Feature Extraction Software version 10.7.3.1 (Agilent Technologies). Poor-quality hybridizations (quality control >0.2) were disregarded [23]. CNAs were identified with Nexus software 7.0 (BioDiscovery, Hawthorne, CA, USA) using the FASST2 segmentation algorithm, which is an approach based on the Hidden-Markov model that uses the log_2_ ratio values of the probes for CNA calling. We applied the following settings: a minimum of five consecutive affected probes (effective resolution of ∼200 kb for CNA calling), a significance threshold set at 10^−8^, and threshold log_2_ ratio Cy3/Cy5 of 0.3 and 1.4 for gain or high copy gain (named amplicons), respectively, and −0.3 and −1.1 for loss and homozygous loss, respectively. Copy number variants reported in the Database of Genomic Variants (DGV, http://projects.tcag.ca/variation/), CNAs smaller than 50 kb, and data from the sex chromosomes were excluded. Gene annotation was performed according to the GRCh37 using the University of California Santa Cruz Genome Browser (http://genome.ucsc.edu/cgi-bin/hgGateway). The smallest common regions of recurrent aberrations were obtained by implementing the global frequency statistical approach of the Significance Testing for Aberrant Copy Number (STAC) method [24]. The parameters had a minimum differential threshold of 25 % and had *p* values of *p* ≤ 0.05 for LVI and *p* ≤ 0.01 for histologic grades. The CNA data of each breast tumor group were evaluated for number, length, and types of CNAs (gains, losses, high copy gains, and homozygous losses), and statistical analyses were performed using GraphPad Prism v5.0 (Mann-Whitney test to compare two unpaired groups and Kruskal-Wallis test to compare three unpaired groups).

### Copy number validation by real-time quantitative PCR

Real-time quantitative PCR (real-time qPCR) was performed on three genes (*ADAMTS3 F*:*CGTAGAAAGCCTTTGGG*, *R*:*GGTGCATGATGGAACG*; *HSD17B12 F*:*CCCTTTAAGCCATTCCG*, *R*:*GCCAATATTCAAACCGAGC*, and *RERGL F*:*CCCCACAAAGTTCCTTC*, *R*:*CTCATCTGCTCTGAAACTGG*) using the SYBR Green system (Applied Biosystems, Foster City, CA, USA) on a 7500 System apparatus (Applied Biosystems, Foster City, CA, USA) with reference DNA (Promega) for copy number calibration. Values were normalized based on data from the *GAPDH* (12p13) and *P2RX7* (12q24) genes, which were not affected by copy number changes in these groups of tumors. Duplicates were analyzed using the comparative 2-ΔΔCt cycle threshold method [25]. Values in the range of 0.8–1.2 indicate two copies, <0.6 indicates copy number loss, and >1.4 was considered a gain.

### Microarray gene expression analysis

A subgroup of 32 breast carcinomas was evaluated for gene expression based on cDNA microarrays using a 244K custom platform (Agilent Technologies, Santa Clara, CA, USA). Samples were hybridized following the manufacturer’s protocol. Scanned images were processed using Feature Extraction Software version 10.7.3.1. The identification of genes differentially expressed was performed using the Agilent GeneSpring GX 12.1 software after subtracting background and filtering features flagged as not positive, not significant, or not uniform in normalization (normalization to the 65th percentile shift per array, median across all samples per transcript). To identify differentially expressed genes, we used the *t* test (unpaired with 5 % false-discovery ratio correction) considering a fold change of |2| with *p* ≤ 0.05. The genes belonging to the PAM50 list [26] were excluded from this analysis since their expression profiles predict intrinsic molecular subtypes of breast cancer and, consequently, could interfere with gene changes differentiating tumors according to histologic grades or lymphovascular invasion status. Ingenuity Pathway Analysis (IPA) software was used for the in silico analysis of the sets of differentially expressed genes. Interaction networks were obtained using a core analysis tool, which considered the Ingenuity Knowledge Base (genes + endogenous chemicals), human species, breast cancer cell lines, and all types of tissues and primary cells. The selected networks generated by the program contained at least 40 % of the set of differentially expressed genes identified in the study.

## Results

**Fig. 1:**
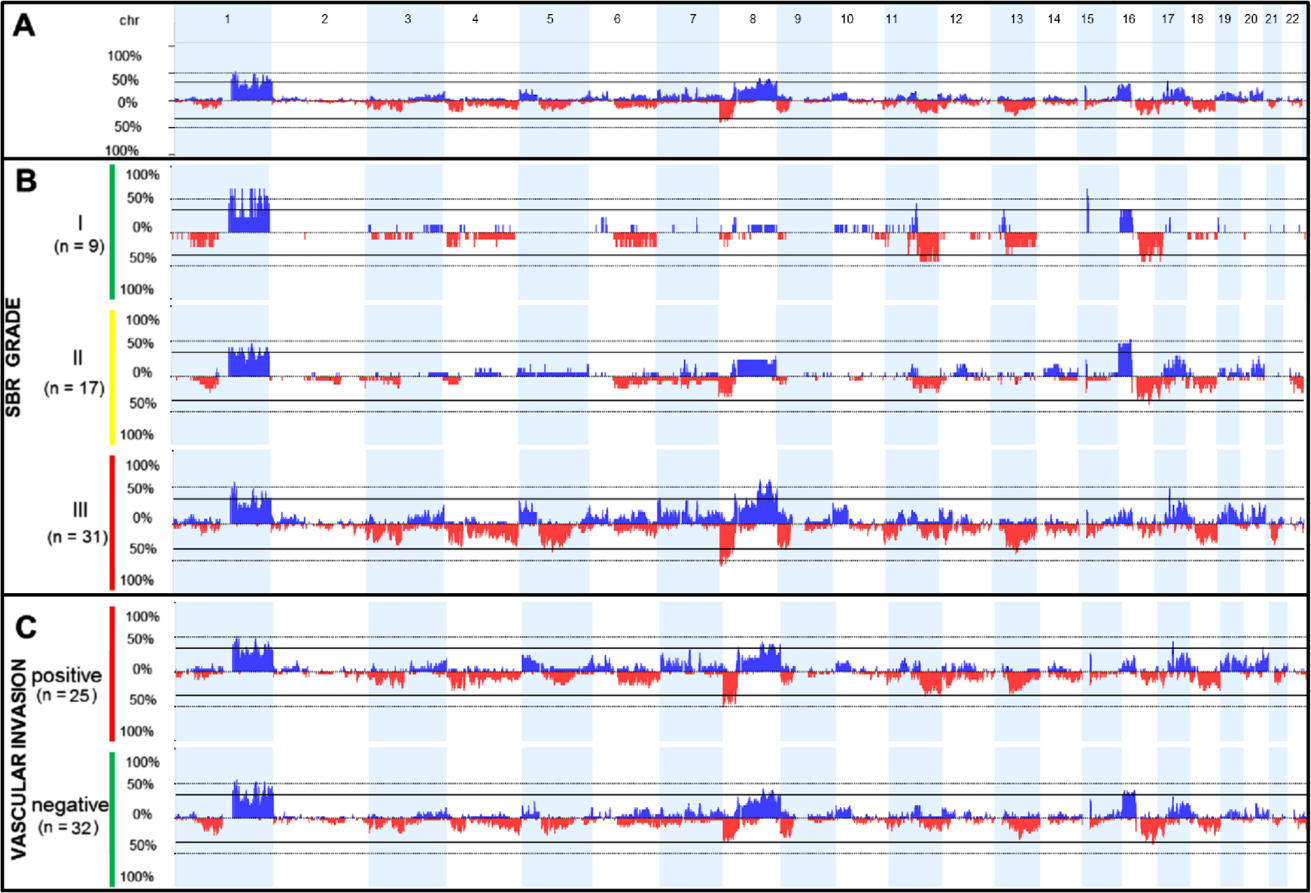
Array-CGH genomic profile exhibiting the frequency of DNA copy number aberrations (CNAs) in the group of invasive ductal carcinomas of the breast. The *x*-axis represents the genomic positions of chromosomes 1p to 22q, and the *y*-axis denotes the percentage of gains (*plotted in blue* above the 0 % baseline) and losses (*plotted in red* below the 0 % baseline) in all selected samples at the specified genomic location (images adapted from the Nexus Copy Number 7.0 software, BioDiscovery). *chr* indicates the chromosome, and *n* is the number of samples in each group. **a** The genomic profile of CNAs (gains and losses) of all 57 invasive ductal carcinomas of the breast. **b** CNA profile of breast tumors grouped according to histologic grade (I, II, and III). **c** CNA profile of breast tumors grouped according to lymphovascular invasion status.

Table 1 summarizes the frequencies of the clinical categories in the group of 57 carcinomas of the breast. Of these cases, 21 % were classified as triple-negative (estrogen receptor (ER)-, progesterone receptor (PR)-, and human epidermal growth factor receptor 2 (HER2)-negative tumors) [27]. A total of 2,857 CNAs were identified in all of the breast tumors profiled (mean of ∼50 CNAs per sample). The summary of the CNA results and the full array-CGH data can be found in Supplementary Tables S2 and S3, respectively. A complex pattern of multiple chromosomal gains and losses was identified in all samples, the most frequent being gains of chromosomes 1q, 8q, 16p, and 17q and losses affecting 8p, 9p, 11q, 13q, 16q, and 18q (Fig. 1a).

**Table 1:** Clinical characteristics of the group of 57 primary invasive ductal carcinomas of non-special type of the breast

Clinical variable	Category	Number (%)
LVI^a^	Positive	25 (43.9)
	Negative	32 (56.1)
SBR^b^	I	9 (15.8)
	II	17 (29.8)
	III	31 (54.4)
Clinical stage	I	9 (15.8)
	II	21 (36.9)
	III	26 (45.6)
	IV	1 (1.7)
Axillary lymph node	N0	20 (35.1)
	N1	19 (33.3)
	N2	10 (17.5)
	N3	8 (14.0)
Estrogen receptor	Positive	33 (57.9)
	Negative	24 (42.1)
*ERBB2*	Positive	5 (8.8)
	Negative	52 (91.2)
Triple-negative	Yes	12 (21.0)
	No	45 (79.0)

^a^Lymphovascular invasion

^b^SBR grade, Scarff-Bloom Richardson graduation system (histological grade)

We performed a statistical comparison between subgroups of breast tumor samples according to the presence of lymph node metastasis: tumors from patients without lymph node metastasis (N0 = 20 samples) and tumors from patients harboring lymph node metastasis at diagnosis. Significant differences were not detected.

### Total CNAs and their distribution and type according to histological grade

We compared different tumor subgroups according to histologic grades to assess the total number of CNAs and the CNA distribution with regard to the different types of events (gain, high copy gain, loss, homozygous loss). These results are detailed in Table 2.

**Table 2:** Total number of DNA copy number aberrations (CNAs), their distribution in each different CNA class (gain, loss, high copy gain, homozygous loss), and genomic lengths according to histologic grades (SBR) and lymphovascular invasion status (LVI)

Group	Total events (median)	Gains (median)	Losses (median)	High copy gains (mean)	Homozygous losses (mean)	Mean size (Mb) (median)
Breast carcinomas (*n* = 57)	2,857 (38.0)	1,477 (23.0)	1,245 (16.0)	122 (2.1)	13 (0.2)	70.2 (27.3)
SBR I (*n* = 9)	290 (21.0)	128 (12.0)	155 (9.0)	7 (0.8)	0 (0)	10.0 (27.0)
SBR II (*n* = 17)	606 (20.0)	315 (10.0)	241 (9.0)	44 (2.6)	6 (0.3)	16.2 (26.6)
SBR III (*n* = 31)	1,961* (60.0)	1,034* (33.0)	849* (25.0)	71 (2.3)	7 (0.2)	48.9* (27.3)
LVI positive (*n* = 25)	1,289 (38.0)	676 (25.0)	558 (14.0)	51 (2.0)	4 (0.2)	33.1 (26.6)
LVI negative (*n* = 32)	1,568 (41.5)	801 (22.5)	687 (17.5)	71 (2.2)	9 (0.3)	40.0 (27.6)

*p < 0.05 (statistically significant at this level in the comparison between different SBR grades, Mann-Whitney test)

**Fig. 2:**
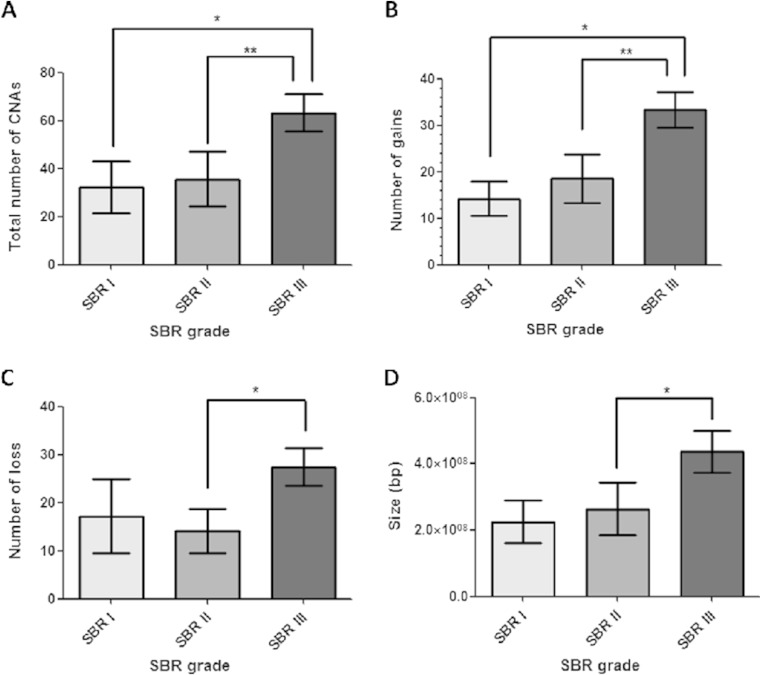
Statistical analysis comparing the DNA copy number aberration (CNA) events between different breast carcinoma groups according to histologic grades. *Asterisks* indicate statistical significance. **a** *Bar graph* of the total number of CNAs showing significant differences between tumor grades I and II compared to grade III. **b** *Bar graph* of the number of gain events showing significant differences between tumor grades I and II compared to grade III. **c** *Bar graph* of the number of loss events showing significant differences between tumor grades II compared to grade III. **d** *Bar graph* of the genomic alteration sizes of breast tumors showing significant differences between grade II tumors compared to grade III

Grade III tumors showed a statistically higher total number of CNAs compared to grade I (*p* = 0.045) and grade II (*p* = 0.006) tumors (Mann-Whitney test, Fig. 2a). Additionally, grade III group had a higher number of gains (*p* = 0.007, Fig. 2b) and losses (*p* = 0.0148, Fig. 2c) compared to low-grade tumors (Mann-Whitney test), and the CNAs were larger than those detected in the grade II group (*p* = 0.0498, Mann-Whitney test; Fig. 2d).

In a visual inspection, grade III tumors exhibited a wider range of chromosomal aberrations than grades I and II. In particular, CNAs detected preferentially or almost exclusively in this group included the following: gains affecting 5p, 8q, 10p, 17q12 (*ERBB2*), and 19 and losses at 3p, 4, 5q proximal, 8p, 9p, 11p, 18q, and 21 (Fig. 1b).

Using the STAC statistical analysis, we detected the smallest common regions of aberrations associated with histologic grade III. Peaks of genomic regions preferentially present in grade III tumors (minimum threshold of 25 %) were detected in several chromosomes, including peak gains at 5p15, 7p22, 8q22, 8q24, 10p12.1, 19q12, and 20p13 and peak losses at 4p13p12, 4q34q35, 9p21, 5q11q23, 12p12, and 21q21 (Supplementary Table S4).

In our samples, the manual curation of CNA data from each tumor sample revealed that the most frequent alteration identified both in grades I (33.3 %) and II (52.9 %) was a 16p gain, which was detected only in 15.2 % of the grade III samples (Fig. 1b, Supplementary Table S4). All breast tumors harboring the 16p gain were found to be positive for estrogen receptor.

### Total CNAs and their distribution and type according to LVI status

We also compared the CNA profile of breast tumors according to LVI status and the results are summarized in Table 2. We did not detect statistically significant differences between LVI-positive and LVI-negative groups (*p* value >0.5 in all comparisons) regarding the total number of CNAs, the CNA distribution (number of gain, high copy gain, loss, homozygous loss), and genomic sizes.

Although we did not detect evidence of an increased genomic instability in LVI-positive breast tumors, some specific rearrangements could be detected at a slightly higher frequency in this group compared to the LVI-negative group, such as gains at 5p, 17q12 (*ERBB2*), and 19, and losses affecting 8p, 11q, 18q, and 21 (Fig. 1c).

Using the STAC analysis, we detected more frequently associated with LVI (minimum of 25 % of the samples) two deletions peaks at 11q24.1 and 11q24.3, encompassing the *OR6M1* and *ARHGAP32* genes, respectively, whereas the 16p gain and a 16q23 loss were detected at a higher frequency in tumors without LVI (Supplementary Table S5). In fact, the manual curation of CNA data from each tumor sample revealed that the 16p gain could be detected in about 40 % of the LVI-negative group compared to 24 % of cases presenting LVI. Considering that all these breast tumor samples carrying 16p gains were found to be positive for estrogen receptor, we performed a statistical correction (logic regression) which showed that the 16p gain is a marker independently associated with LVI-negative status.

### Small-scale rearrangements: genomic amplifications and homozygous losses

We investigated the frequency of six known breast cancer amplicons [28] excluding 17q12 (*ERBB2*), and all of them were frequently detected in grade III tumors as genomic gains (Table 3). The 19q12 gain was detected exclusively in grade III tumors (one amplification and nine copy number gains), all of them negative for estrogen receptor. The smallest common region affected by this 19q12 gain was restricted to a ∼537 kb segment at chr19:29,777,917-30,315,215 (GRCh37) in which only six genes are mapped, including *CCNE1*. Regarding the LVI status, the majority of the tumors carrying the 20q13.2 gain (62.5 %) were positive for LVI.

**Table 3:** Frequency of gains affecting well-known breast cancer amplicons in high-risk breast carcinomas (histologic grade III and lymphovascular invasion positive)

Known amplicon	Major gene	Total frequency (*n* affected)	Frequency among histologic grade III samples	Frequency among lymphovascular invasion positive samples
8q24	*MYC*	38.6 % (22)	**81.8 %**	45.4 %
8p12	*ZNF703*/*FGR1*	31.6 % (18)	**72.2 %**	44.4
8p12	*FGFR1*	29.8 % (17)	**64.7 %**	47.1 %
19q12	*CCNE1*	17.5 % (10)	**100 %**	**55.5 %**
20q13.2	*ZNF217*	14.0 % (8)	**62.5 %**	**62.5 %**
11q13	*CCND1*	14.0 % (8)	**50.0 %**	25.0 %

In bold are indicated amplicons that were frequently detected (≥50 %) in tumors of each category

**Fig. 3:**
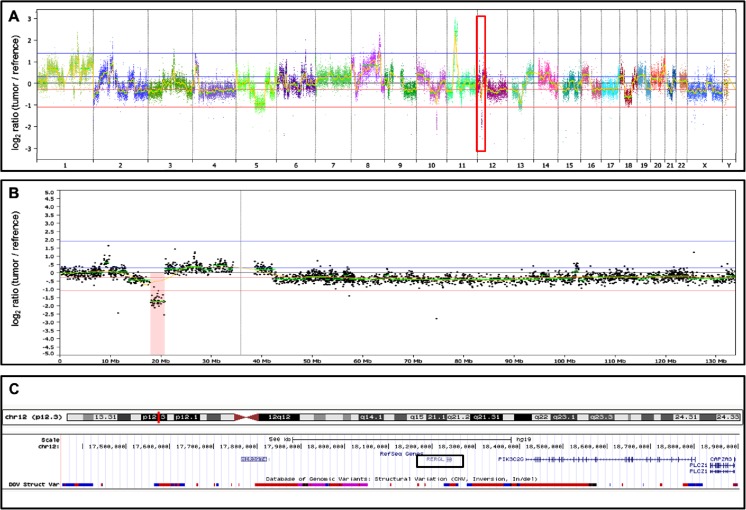
Homozygous deletion affecting a 1.5 Mb genomic region at 12p13.3, detected in a high-risk breast tumor (histologic grade III with lymphovascular invasion, MIC82). **a** Array-CGH genomic profile of the breast carcinoma -the deletion at chromosome 12 is indicated by a *red box*. **b** Array-CGH profile of chromosome 12, highlighting the ∼1.5 Mb deletion at 12p13.3 (in *red*). **c** The affected genomic segment at 12p13.3 is depicted by the UCSC Genome Browser; this region encompasses five genes, including *RERGL* (marked by a *black box*) and *PIK3C2G*

In addition to the abovementioned known breast cancer amplicons, we detected three small-scale rearrangements (< 4 Mb) not previously reported, each of them in a different high-risk tumor (grade III positive for LVI): two amplifications, one at 4q13.3 (*chr4*:*72*,*209*,*104-73*,*455*,*050*) and the other at 11p11.2 (*chr11*:*43*,*830*,*876-46*,*190*,*463*); and one homozygous deletion at 12p12.3 (*chr12*:*17*,*950*,*780-20*,*811*,*443*). Genes mapped within the two amplified regions were selected for further copy number validation (Supplementary Figure S1). Figure 3 displays the homozygous 12p12.3 deletion encompassing the *RERGL* and *PIK3C2G* genes, among others.

The 11p11.2 genomic amplification encompasses, among others, the *HSD17B12* gene. In our cohort, we detected *HSD17B12* gains in association with gains of the *COX2* gene, in a possible 1q31/11p11 co-amplification pattern, here observed in ER-negative high-grade breast tumors.

### Copy number status of previously reported breast cancer driver genes

**Fig. 4:**
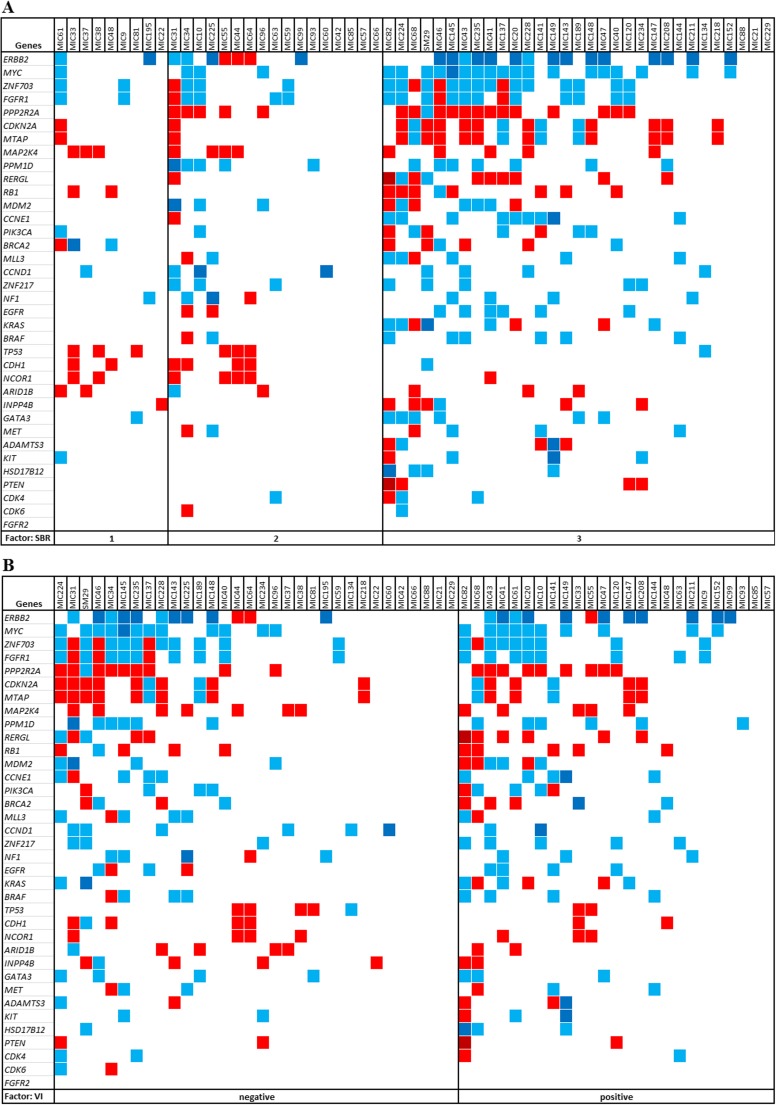
Copy number changes of a panel of 36 genes in all 57 breast tumor samples grouped according to SBR grades or lymphovascular invasion status. The copy number status of 33 breast cancer driver genes and 3 genes (*rows*) identified in the present study (*ADAMTS3*, *HSD17B12*, and *RERGL*) is depicted for each tumor sample (*columns*) by *colored squares*: gains and amplifications are shown in *blue* and *dark blue*, respectively; losses and homozygous losses are *red* and *dark red*, respectively. **a** Schematic view showing the pattern of copy number changes of the panel of 36 genes in breast tumor samples grouped according to histologic grade. **b** Schematic view showing the pattern of copy number changes of the panel of 36 genes in breast tumors grouped according to lymphovascular invasion status

We scrutinized the copy number status of 33 driver cancer genes reported as recurrently affected by copy number changes in breast cancer [11, 17, 29–32], which includes the major genes of the aforementioned amplicons. We also examined the copy number status of three genes highlighted in the present study (*ADAMTS3*, *HSD17B12*, and *RERGL*). Different CNA frequencies affecting these genes were observed according to histologic grade and LVI status (Fig. 4). A panel of 16 genes exhibited copy number changes at higher frequencies in high-grade tumors compared to low-grade samples (losses affecting *PPP2R2A*, *CDKN2A*, *MTAP*, *RERGL*, *RB1*, *PTEN*, *INPP4B* and gains of *CCNE1*, *ERBB2*, *EGFR*, *FGFR1*, *GATA3*, *MYC*, *HSD17B12*, *ZNF703*, and *ZNF217*; Fig. 4a); conversely, copy number losses of *TP53*, *CDH1*, and *NCOR1* were detected preferentially in low-grade breast tumors. Furthermore, *ERBB2* gains and *RERGL* losses were more frequent in the presence of LVI (Fig. 4b).

### Gene expression profiling

**Fig. 5:**
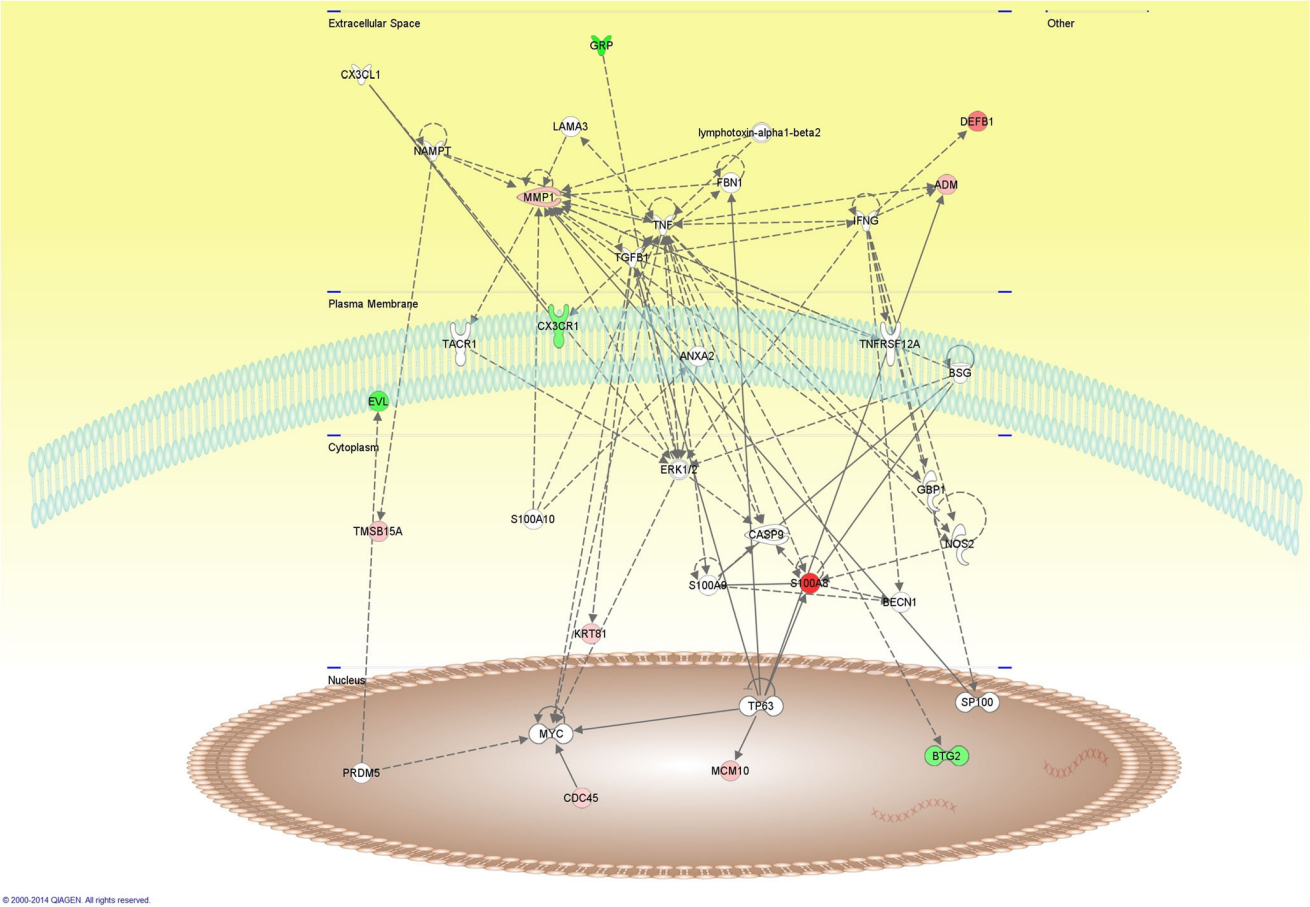
Cellular diagram of the network created by the Ingenuity Pathway Analysis based on interactions of the set of differentially expressed genes detected in histologic grade III breast tumors compared to grade I and grade II tumors. *Red* and *green* nodes represent, respectively, upregulated or downregulated genes (the intensity of the colors indicating the degree of deregulation) in grade III breast tumors in comparison to grade I and II tumors (Table 4); *uncolored nodes* represent genes automatically included in this network because they are biologically linked to the others based on scientific evidence. The functional categorization of this network was Cellular Movement, Hematological System Development and Function, Immune Cell Trafficking, with the *S100A8* gene playing a central role

Table 4 displays the top differentially expressed genes of the breast tumor groups with their respective fold-change values (full differential expression analysis can be found in Supplementary Tables S6 and S7). Histological grade III tumors exhibited 12 up- and 13 downregulated genes compared to low-grade tumors (*p* value <0.001 and fold change of |5|). We investigated the copy number status of the detected deregulated genes. The following five genes displayed a CNA pattern concordant with the expression level in >15 % of the grade III tumors: *S100A8* and *MCM10* genes (upregulated) and *GRP*, *CX3CR1*, and *FAM198B* (downregulated). We used the network analysis tool of IPA software to reveal regulatory interconnections among these genes. The network with the highest score (26) connected 12 of the deregulated genes with 23 additional ones, showing an enrichment of the categories of Cellular Movement, Hematological System Development and Function, Immune Cell Trafficking (Fig. 5), in which the *S100A8* gene plays a central role.

**Table 4:** Top differentially expressed genes (positive fold-change values indicating upregulation and negative fold-change values indicating donwregulation) identified between histologic grade III breast tumors and histologic grade I+II breast tumors (left) and between breast tumors positive or negative for lymphovascular invasion (right)

Histologic grade III^a^				Lymphovascular invasion positive^b^			
Gene	Cytoband	Fold-change	*p* value	Gene	Cytoband	Fold-change	*p* value
*S100A8*	1q21.3	22.0278	0.001	*C19orf33*	19q13.2	4.4608	0.021
*DEFB1*	8p23.1	14.2762	0.001	*LGALS7B*	19q13.2	3.5713	0.007
*ADM*	11p15.4	7.0605	0.001	*CPE*	4q32.3	3.0436	0.011
*MMP1*	11q22.2	6.7464	0.001	*AGBL2*	11p11.2	2.9353	0.028
*MCM10*	10p13	6.4085	0.001	*ARSG*	17q24.2	2.7475	0.044
*TMSB15A*	Xq22.1	6.2266	0.001	*UMOD*	16p12.3	2.6808	0.025
*SOX11*	2p25.2	5.5269	0.001	*C13orf31*	13q14.11	2.6631	0.026
*YBX2*	17p13.1	5.3408	0.001	*MYCBPAP*	17q21.33	2.5776	0.032
*KRT81*	12q13.13	5.3214	0.001	*CXXC4*	4q24	2.5767	0.044
*E2F8*	11p15.1	5.2362	0.001	*MED1*	17q12	2.5187	0.033
*NANOS1*	10q26.11	5.1092	0.001	*SHISA5*	3p21.31	2.5084	0.002
*CDC45L*	22q11.21	5.0596	0.001	*GFRA1*	10q25.3	−4.4772	0.021
*GRP*	18q21.32	−8.9074	0.001	*MFI2*	3q29	−4.0174	0.029
*CYP4X1*	1p33	−8.1935	0.001	*TBX21*	17q21.32	−3.8633	0.038
*SUSD3*	9q22.31	−8.0443	0.001	*KRT15*	17q21.2	−3.6634	0.016
*ABAT*	16p13.2	−7.3311	0.001	*NFASC*	1q32.1	−3.3951	0.022
*EVL*	14q32.2	−6.9978	0.001	*FZD5*	2q33.3	−3.1834	0.008
*KLHDC9*	1q23.3	−6.9593	0.001	*TCF7L1*	2p11.2	−3.0653	0.047
*C6orf211*	6q25.1	−6.9305	0.001	*MYBPC2*	19q13.33	−2.8301	0.021
*NEFH*	22q12.2	−6.3975	0.001	*CRABP1*	15q25.1	−2.7727	0.017
*CX3CR1*	3p22.2	−5.3859	0.001	*CIB2*	15q25.1	−2.5553	0.018
*SMOC2*	6q27	−5.3324	0.001	*DUSP3*	17q21.31	−2.5391	0.001
*BTG2*	1q32.1	−5.3206	0.001				
*FAM198B*	4q32.1	−5.0859	0.001				
*CCDC74B*	2q21.1	−5.0592	0.001				

^a^Histological grade III: *p* value <0.001 and fold change of |5|

^b^Lymphovascular invasion positive: *p* value <0.05 and fold change of |2.5|

Breast tumors with LVI exhibited 11 upregulated and 11 downregulated genes compared to negative tumors (*p* value <0.05 and fold change of |2.5|). The following four upregulated genes exhibited a concordant CNA pattern in tumors with LVI: *UMOD* (gains) and *ARSG*, *MYCBPAP*, and *MED1* (gains and high-copy gains). The 22 deregulated genes were evaluated using the network analysis tool of IPA software. The more relevant network (score 12) connected seven of the differentially expressed genes with additional 28 genes, which showed an enrichment of the categories of Cellular Development, Cellular Growth and Proliferation, Hematological System Development and Function. In this network, the *MED1* gene appears to play a central role (Supplementary Figure S2).

### Summary of the relevant genomic regions and selected genes

Table 5 presents a summary of the relevant genomic regions and genes revealed in this work in association either with histologic grade III tumors or with LVI. Data include some of the significant recurrent CNAs, highlighting relevant gene content, as well as three newly detected small-scale rearrangements, and differentially expressed genes possibly deregulated by copy number changes.

**Table 5:** Summary of the relevant genomic regions and genes revealed in this work in association either with histologic grade III or with lymphovascular invasion

Relevant gene(s)	Chromosomal alteration	Clinical association
*MYC*	8q24 gain	Preferentially detected in grade III samples
*MTAP*	9p21 loss	Preferentially detected in grade III samples
*COX6C*	8q22 gain	Preferentially detected in grade III samples
*PMS2*	7p22 loss	Preferentially detected in grade III samples
*ERCC8*, *XRCC4*, *FBXL17*, *MEF2C*	5q11q13 loss	Preferentially detected in grade III samples
*LMO3*	12p12.3 loss	Preferentially detected in grade III samples
*CCNE1*, *LOC284395*, *VSTM2B*, *POP4*, *PLEKHF1*, *C19orf12*	19q12 minimum common region of gain	Exclusively detected in grade III negative for estrogen-receptor samples
*ADAMTS3*	4q13.3 amplification	Detected in a grade III LVI-positive sample
*HSD17B12*	11p11.2 amplification	Detected in a grade III LVI-positive sample
*RERGL*, *PIK3C2G*	12p12.3 homozygous deletion	Detected in a grade III LVI-positive sample
*ARHGAP32*	11q24.3 loss	Preferentially detected in LVI-positive samples
*S100A8*, *MCM10*	–	Concordant pattern of upregulated gene expression and copy number gains in grade III samples
*GRP*, *CX3CR1*, *FAM198B*	–	Concordant pattern of downregulated gene expression and copy number losses in grade III samples
*UMOD*, *ARSG*, *MYCBPAP*, *MED1*	–	Concordant pattern of upregulated gene expression and copy number gains/high gains in LVI-positive samples
*Several*	16p gain	Preferentially detected in both low-grade and LVI-negative samples (exclusively estrogen-receptor-positive tumors)

## Discussion

Increased numbers of CNAs are expected to be associated with tumor aggressiveness [12]; accordingly, we detected a particular augmentation of the number of genomic gains in histologic grade III breast tumors. Despite the fact that LVI is also associated with high-risk breast cancer, no differences in the total number of CNAs were detected in our study considering the LVI status. Taken together, our data corroborate previous studies showing an accumulation of genomic alterations in more aggressive breast tumors, although it also suggests that vascular invasion is a prognostic marker not related to increased genomic instability.

### CNA pattern according to histological grade

Grade III tumors exhibited a wide range of genomic aberrations previously reported for this category [11, 12]; the previously reported 19q12 amplification [17] was detected in only one grade III tumor, although gains affecting 19q12 were identified in additional nine grade III samples, which allowed us to narrow the critical region to a ∼537 kb segment. It is noteworthy that all grade III tumors carrying the 19q12 gain were found to be negative for estrogen receptor, a finding that corroborates the link with poor outcome [11, 28].

Some regions statistically associated with grade III tumors in our study harbor genes either frequently amplified in aggressive breast cancer such as the oncogenes *MYC* (8q24) and *CCNE1* (19q12) or already reported as deleted in breast tumors such as *MTAP* (9p21) [30]. The 8q22 gain is reportedly related to poor prognosis and chemoresistance possibly due to the activation of *MTDH* [33], although the 8q22 segment here detected did not harbor this gene and included the cancer gene *COX6C*, which is involved in oxidative metabolism, upregulated in prostate tumors, and fusioned to *HMGIC* gene in uterine leiomyomas [34, 35]. Conversely, some of the detected regions in the present work are not strongly related to breast cancer; recurrent losses affecting 7p22 (segment encompassing, among others, the *PMS2* gene) and 5q11-q13 (*ERCC8* and *XRCC4*) could suggest the involvement of DNA repair pathways in cancer progression. The 5q loss also includes *FBXL17*, a gene reported to be a biomarker for breast cancer resistance [36], and *MEF2C*, which was previously identified in association with breast cancer invasion [37]. The loss at 12p12.3 includes *LMO3*, a key gene implicated in the onset and progression of several cancers [38].

Consistent with the literature [12, 18, 39], the 16p gain was detected here preferentially in low-grade tumors (33.3 % of grade I samples and 52.9 % of grade II samples), and all of them were estrogen-positive samples.

### CNA pattern according to LVI status

Although the LVI-positive tumors were not associated with an increased genomic instability compared to LVI-negative tumors, they showed a few genomic alterations detected at a slightly higher frequency than the negative group, such as gains at 5p, 17q12 and 19, and losses at 8p, 11q, 18q, and 21. The most interesting alteration was an 11q24.3 loss harboring *ARHGAP32*, a gene earlier reported to be involved in fusion events in breast cancer cell lines and to participate in autophagy processes [40–43]. Additionally, the 16p gain was also preferentially associated with the absence of LVI (40 % of LVI-negative tumors), which reinforces this CNA as a marker of good prognosis [12, 18, 39].

### Copy number status of known driver genes and new genes proposed for breast cancer

Known breast cancer amplicons were detected as gains either exclusively (19q12) or predominantly in grade III tumors [15, 44]. The 20q13.2 amplicon, previously associated with poor outcome [30, 44–47], was related in our study both to high-grade and LVI-positive tumors. We identified three new focal rearrangements in grade III/LVI-positive breast tumors: 4q13 and 11q11 amplifications (harboring the *ADAMTS3* and the *HSD17B12* genes, respectively) and a homozygous deletion at 12p12.3 (containing, among others, the *RERGL* gene). These highlighted regions because of the small size and the amplitude of the copy number changes indicate potential driver genes for breast cancer. The *ADAMTS3* gene belongs to the ADAMTS metalloproteinase family that has been implicated in tumor progression [44–46]. Overexpression of *HSD17B12* was recently associated with *COX2* (1q31) overexpression in breast carcinomas [47], and in our cohort, *HSD17B12* gains were detected in association with *COX2* gains, which may indicate a new 1q31/11p11 co-amplification pattern in ER-negative high-grade breast tumors. However, functional studies are necessary to validate the biological relevance of the emphasized genes.

Additionally, we assembled a panel of genes exhibiting differences in the frequency of copy number changes between high-grade and low-grade breast tumors groups (see Table 5), including two of those genes here reported (*RERGL* and *HSD17B12*). Finally, the combination of frequencies of copy number changes of two genes was detected in LVI-positive breast cancer group: gains of *ERBB2* and losses of *RERGL*.

### Gene expression profiling

Concerning the gene expression pattern of grade III breast tumors, the *S100A8* gene, the top upregulated gene in grade III compared to low-grade tumors, appeared to be a central gene in the main regulatory network. This gene can act as a potent amplifier of inflammation in autoimmunity as well as of cancer development and metastasis [48–50]. *S100A8* overexpression in high-grade breast tumors in comparison to low-grade tumors has been previously reported [51] and associated with poor prognosis, poor tumor differentiation, LVI, and node metastasis when co-expressed with *S100A9* [52]. Another promising candidate gene for breast cancer aggressiveness is *MMP1*, which belongs to a matrix metalloproteinase family associated with cell growth, metastasis, and the progression of different neoplasias, including breast cancer [53–56]. A recent paper by our group disclosed another gene of the same family, *MMP2*, as a key player in the progression of ductal carcinomas of the breast [57].

Some of the genes with the highest differential expression (fold change >|5| for histological grade, fold change >|3| for LVI) exhibited direct correlations between copy number changes and expression levels, such as gains affecting *S100A8* and *MCM10*, which suggests that expression levels could be driven by CNAs [17, 58, 59]. We also detected a downregulation pattern for *RERG*, a gene from the family of the abovementioned deleted *RERGL* gene.

Regarding the LVI tumors, the most upregulated gene in the LVI-positive group compared to the negative group was *C19orf33* which is also known as *IMUP* (*immortalization upregulated protein*), a gene previously shown to be overexpressed in lung, colon, and ovarian carcinoma cell lines [60, 61]. Another remarkable upregulated gene was *LGALS7B*, a basal cell marker which enhances metastasis to the lungs and bones in breast cancer [62]. Additionally, the upregulated *MED1* gene, earlier reported in association with metastasis and therapy resistance in breast tumors [63], was uncovered as a relevant gene in a network enriched for genes in the categories of Cellular Growth and Proliferation and Hematological System Development and Function. A concordant pattern of association between CNAs and the expression profile was detected in our study for some genes already implicated in invasion and metastasis, such as gains and amplifications of *MYCBPAP* and *MED1* [63, 64]. Furthermore, downregulated genes in tumors exhibiting LVI included *MFI2*, a gene associated with breast cancer metastasis when downregulated [65].

## Conclusion

Therefore, we could delineate a group of genomic alterations and relevant genes (see Table 5) that seems to be associated with high-grade breast cancer as well as with LVI. We recognize that histologic grade and LVI are easily evaluated in routine histological analysis, but CNA screening upon the diagnosis of low-grade breast tumors could assist in the identification of potentially more aggressive tumors requiring further investigation. Our study has shed some light on the molecular players involved in two highly relevant prognostic factors and may enable a better understanding of the mechanisms of breast cancer aggressiveness.

## Electronic supplementary material

Below is the link to the electronic supplementary material.

Supplementary Figure S1 Real-time qPCR validation of two small-scale rearrangements not previously reported in the literature. The graphs depict the copy number average of three replicates of each investigated gene in the breast tumor sample carrying the copy number alteration detected by array-CGH. (A) Real-time qPCR data showing a high number of copies of the *HSD17B12* gene, confirming the 11p11.2 amplification detected in the *MIC82* breast tumor sample. (B) Real-time qPCR data showing a high number of copies of the *ADAMTS3*, confirming the 4q13.3 amplification detected in the *MIC149* breast tumor sample. (PPTX 53 kb)

Supplementary Figure S2 Cellular diagram of the network created by the Ingenuity Pathway Analysis based on interactions of the set of differentially expressed genes detected in breast tumors positive for lymphovascular invasion compared to tumors without invasion. Red and green nodes represent up-regulated or down-regulated genes, respectively, in breast tumors positive for lymphovascular invasion in comparison to negative tumors (Table 4) (the intensity of the colors indicating the degree of deregulation); uncolored nodes represent genes automatically included in this network because they are biologically linked to the others based on scientific evidence. The functional categorization of this network revealed Cellular Development, Cellular Growth and Proliferation, Hematological System Development and Function; in this network, the *MED1* gene appeared to be a key gene. (GIF 1765 kb)

High Resolution (TIFF 2242 kb)

Supplementary Table S3 (XLSX 928 kb)

Supplementary Table S1 (DOCX 35 kb)

Supplementary Table S2 (DOCX 22 kb)

Supplementary Table S4 (DOCX 17 kb)

Supplementary Table S5 (DOCX 14 kb)

Supplementary Table S6 (DOCX 36 kb)

Supplementary Table S7 (DOCX 21 kb)
