# Supplementary figures and images for: Lymphovascular invasion and histologic grade are associated with specific genomic profiles in invasive carcinomas of the breast
Source: Tumour Biol. 2014 Nov 13;36(3):1835–48. doi: 10.1007/s13277-014-2786-z (PMC4375298; doi:10.1007/s13277-014-2786-z)

## Slide 1
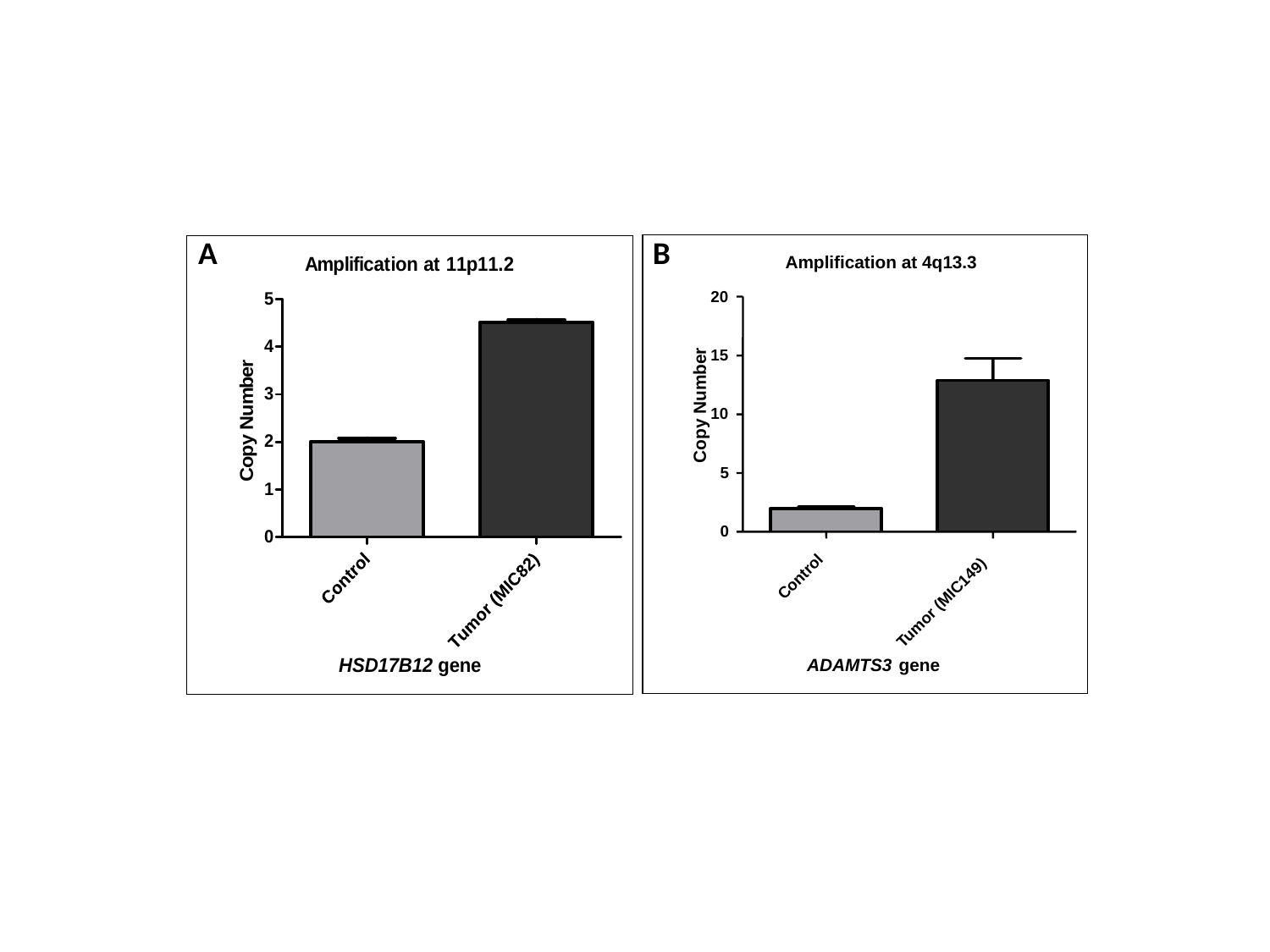

A
B
Amplification at 4q13.3
20
15
Copy Number
10
5
0
Control
Tumor (MIC149)
 gene
ADAMTS3

Supplement: Supplementary file 1 — Real-time qPCR validation of two small-scale rearrangements not previously reported in the literature. The graphs depict the copy number average of three replicates of each investigated gene in the breast tumor sample carrying the copy number alteration detected by array-CGH. (A) Real-time qPCR data showing a high number of copies of the HSD17B12 gene, confirming the 11p11.2 amplification detected in the MIC82 breast tumor sample. (B) Real-time qPCR data showing a high number of copies of the ADAMTS3, confirming the 4q13.3 amplification detected in the MIC149 breast tumor sample. (PPTX 53 kb) [file 13277_2014_2786_MOESM1_ESM.pptx]

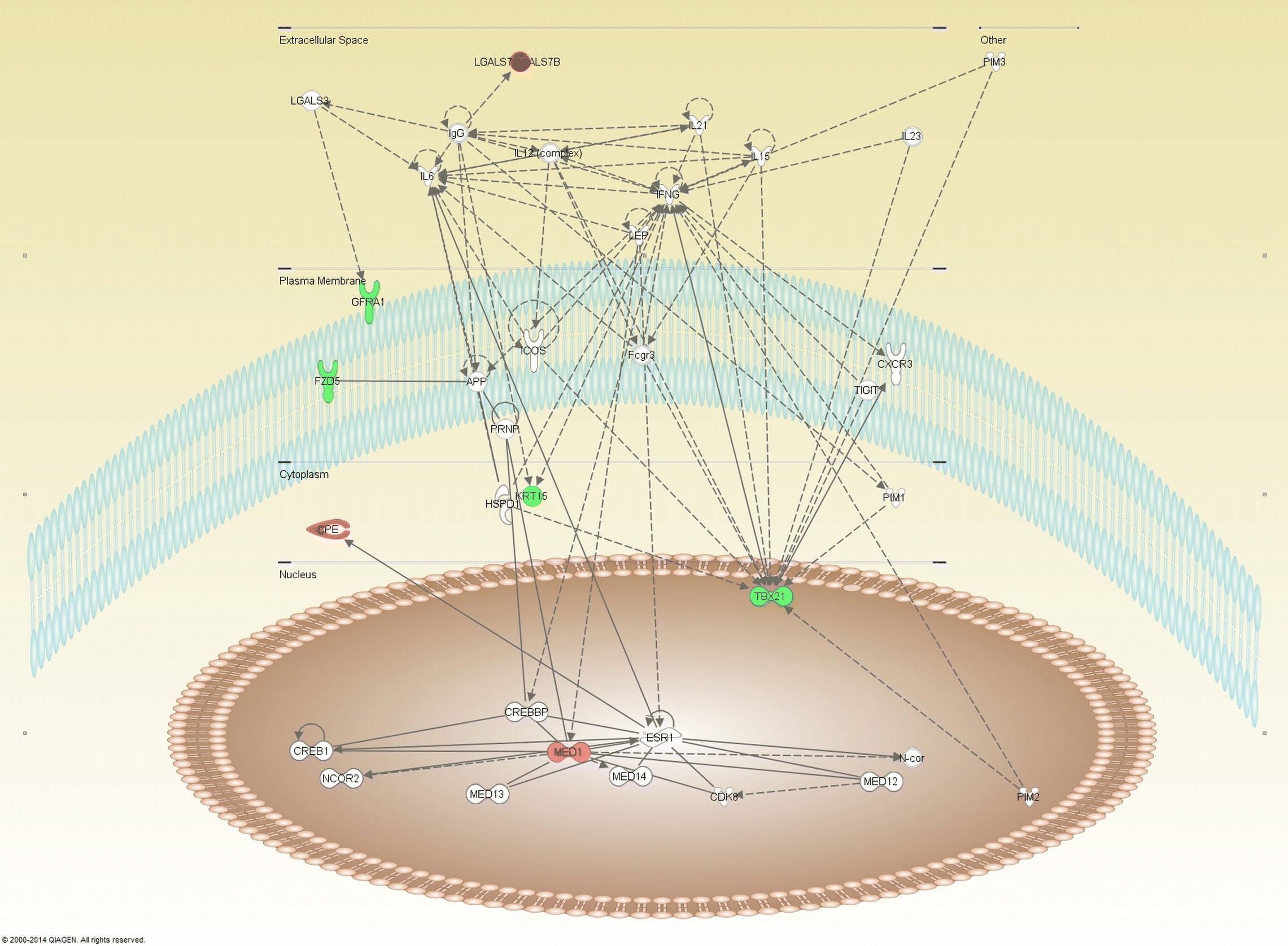

Supplement: Supplementary file 2 — Cellular diagram of the network created by the Ingenuity Pathway Analysis based on interactions of the set of differentially expressed genes detected in breast tumors positive for lymphovascular invasion compared to tumors without invasion. Red and green nodes represent up-regulated or down-regulated genes, respectively, in breast tumors positive for lymphovascular invasion in comparison to negative tumors (Table 4) (the intensity of the colors indicating the degree of deregulation); uncolored nodes represent genes automatically included in this network because they are biologically linked to the others based on scientific evidence. The functional categorization of this network revealed Cellular Development, Cellular Growth and Proliferation, Hematological System Development and Function; in this network, the MED1 gene appeared to be a key gene. (GIF 1765 kb) [file 13277_2014_2786_Fig6_ESM.gif]

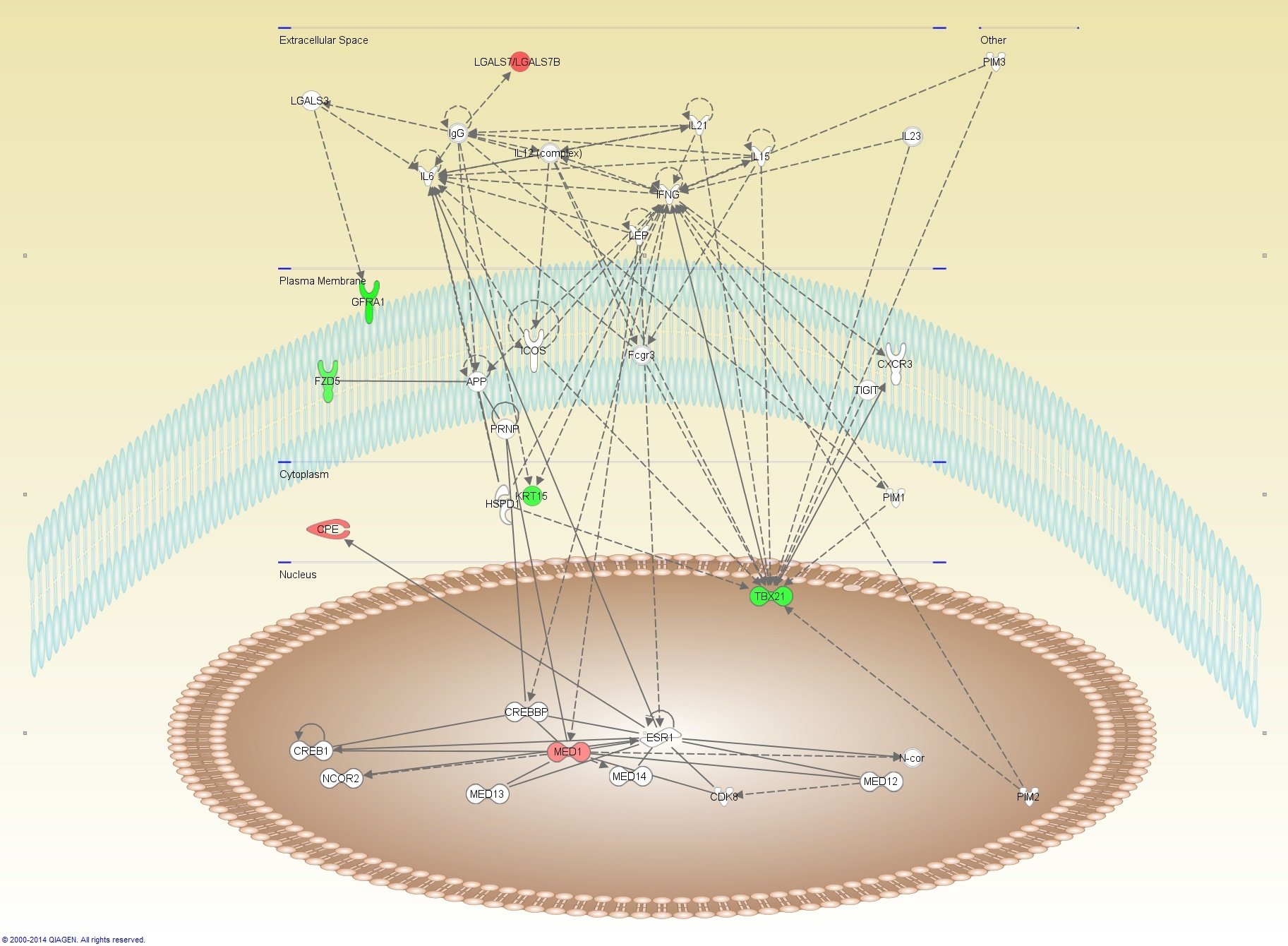

Supplement: Supplementary file 3 — High Resolution (TIFF 2242 kb) [file 13277_2014_2786_MOESM2_ESM.tif]
