# Supplementary material for: Lymphovascular invasion and histologic grade are associated with specific genomic profiles in invasive carcinomas of the breast
Source: Tumour Biol. 2014 Nov 13;36(3):1835–48. doi: 10.1007/s13277-014-2786-z (PMC4375298; doi:10.1007/s13277-014-2786-z)
Supplement: Supplementary file 5 — (DOCX 35 kb) [file 13277_2014_2786_MOESM4_ESM.docx]

**SUPPLEMENTARY TABLES**

**Supplementary Table S1.** Description of clinical and anatomopathologic data of the 57 samples of primary invasive ductal carcinoma of the breast.

| Sample | Stage | TNM^1^ | SBR^2^ | | | | LVI^3^ | PR^4^ | ER^5^ | ERBB2 | | Lymph node* | p53 | ki67 | Tumor size (cm) |
| --- | --- | --- | --- | --- | --- | --- | --- | --- | --- | --- | --- | --- | --- | --- | --- |
|  |  |  | **SBR** | **Mitosis** | **Nucleus** | **Tubule** |  |  |  | **IHQ^6^** | **FISH^7^** |  |  |  |  |
| MIC10 | 3b | T4bN1M0 | 2 | 2 | 2 | 2 | pos | neg | pos | 3+ | - | 7/1 | - | - | 11.0 |
| MIC120 | 3c | T2N3M0 | 3 | 3 | 3 | 3 | pos | pos | pos | neg | - | 19/17 | - | - | 2.3 |
| MIC134 | 2 | T2N1M0 | 3 | 3 | 3 | 3 | neg | neg | neg | neg | - | 20/1 | - | - | 3.8 |
| MIC137 | 2 | T1cN1M0 | 3 | 3 | 3 | 3 | neg | neg | neg | neg | - | 23/1 | - | - | 1.3 |
| MIC141 | 2b | pT2pN1 | 3 | 3 | 3 | 3 | pos | neg | neg | neg | - | 26/3 | pos | - | 5.0 |
| MIC143 | 1 | pT1cN0M0 | 3 | 3 | 3 | 3 | neg | neg | neg | 2+ | HER2/chr17=3 | 17/0 | - | - | 1.6 |
| MIC144 | 2b | T2N3M0 | 3 | 3 | 3 | 3 | pos | neg | neg | neg | - | 46/34 | pos | - | 3.5 |
| MIC145 | 2 | T2N0M0 | 3 | 2 | 3 | 3 | neg | neg | neg | 2+ | HER2/chr17=5 | 32/0 | - | - | 2.7 |
| MIC147 | 3a | T2N2M0 | 3 | 3 | 3 | 3 | pos | neg | neg | 3+ | - | 10/4 | - | - | 2.3 |
| MIC148 | 1 | T1cN0M0 | 3 | 2 | 3 | 3 | neg | neg | neg | 3+ | - | 3/0^#^ | - | - | 1.5 |
| MIC149 | 1 | T1cN2M0 | 3 | 3 | 3 | 3 | pos | neg | neg | 3+ | - | 21/2 | - | - | 1.2 |
| MIC152 | 3 | pT4bpN2M0 | 3 | 2 | 3 | 3 | pos | neg | neg | 3+ | - | 24/6 | - | pos | 2.0 |
| MIC189 | 3a | T2N2M0 | 3 | 3 | 3 | 3 | neg | neg | neg | neg | - | 43/4 | pos | - | 4.2 |
| MIC195 | 1 | T1bN0M0 | 1 | 2 | 2 | 1 | neg | pos | pos | 3+ | - | 8/0 | - | - | 1.0 |
| MIC20 | 3b | T4bN2M0 | 3 | 3 | 3 | 3 | pos | neg | neg | 2+ | - | 38/8 | - | - | 6.0 |
| MIC208 | 4 | T4N1M1 | 3 | 2 | 3 | 3 | pos | neg | neg | 3+ | - | 16/3 | - | - | 5.5 |
| MIC21 | 3b | pT1cN0M0 | 3 | 3 | 2 | 3 | neg | neg | neg | neg | - | 20/0 | - | - | 1.5 |
| MIC211 | 2 | T2N0M0 | 3 | 3 | 3 | 3 | pos | pos | pos | 3+ | - | 2/0^#^ | - | - | 3.0 |
| MIC218 | 2a | T2N0 | 3 | 3 | 3 | 3 | neg | neg | neg | neg | - | 28/0 | - | - | 2.5 |
| MIC22 | 1 | T1cN1M0 | 1 | 1 | 1 | 1 | neg | neg | pos | 2+ | - | 29/3 | pos | - | 1.8 |
| MIC224 | 1 | T2N0M0 | 3 | 3 | 3 | 3 | neg | neg | neg | neg | - | 8/0 | pos | pos | 3.0 |
| MIC225 | 1 | T1cN0M0 | 2 | 2 | 2 | 2 | neg | neg | pos | 3+ | - | 2/0 | - | pos | 2.0 |
| MIC228 | 3a | T2N2M0 | 3 | 3 | 3 | 3 | neg | neg | neg | neg | FISH neg | 34/9 | pos | pos | 3.5 |
| MIC229 | 2 | T1N1M0 | 3 | 3 | 3 | 3 | neg | neg | neg | 2+ | HER2/cr17=2.5 | 15/1 | - | - | 2.0 |
| MIC234 | 2a | T2N0M0 | 3 | 3 | 3 | 3 | neg | neg | neg | neg | - | 36/0 | pos | pos | 3.5 |
| MIC235 | 3 | pT2cpN2M0 | 3 | 3 | 3 | 3 | neg | pos | pos | 2+ | HER2/cr17 >5 | 20/6 | - | - | 2.0 |
| MIC31 | 2a | T2N0M0 | 2 | 1 | 2 | 3 | neg | neg | pos | 2+ | FISH neg | 21/0 | - | - | 4.5 |
| MIC33 | 3b | pT4cN1M0 | 1 | 1 | 2 | 1 | pos | neg | pos | neg | - | 34/1 | - | - | 3.3 |
| MIC34 | 2a | T2N1M0 | 2 | 1 | 3 | 2 | neg | pos | pos | neg | - | 27/1 | - | - | 3.2 |
| MIC37 | 2a | T2N0M0 | 1 | 1 | 2 | 2 | neg | pos | pos | neg | - | 24/0 | pos | - | 3.0 |
| MIC38 | 2a | T2N2M0 | 1 | 1 | 2 | 1 | neg | pos | pos | neg | - | 19/5 | - | - | 2.4 |
| MIC40 | 3b | T4bN1M0 | 3 | 2 | 3 | 3 | neg | pos | pos | neg | - | 16/2 | - | - | 7.0 |
| MIC41 | 1 | T1cN1M0 | 3 | 3 | 2 | 3 | pos | neg | pos | neg | - | 24/2 | pos | - | 1.8 |
| MIC42 | 1 | T1cN0M0 | 2 | 1 | 3 | 3 | neg | pos | pos | neg | - | 23/0 | - | - | 1.5 |
| MIC43 | 3b | T4bN3M0 | 3 | 3 | 3 | 3 | pos | pos | pos | neg | - | 22/17 | pos | - | 6.0 |
| MIC44 | 2a | T2N0M0 | 2 | 1 | 2 | 3 | neg | pos | pos | neg | - | 17/0 | - | - | 6.5 |
| MIC46 | 3c | pT2pN3M0 | 3 | 2 | 3 | 3 | neg | neg | neg | 3+ | - | 31/17 | - | - | 4.4 |
| MIC47 | 3b | T4bN3M0 | 3 | 3 | 3 | 3 | pos | neg | neg | 3+ | - | 21/21 | - | - | 16.0 |
| MIC48 | 3a | T3N2M0 | 1 | 1 | 2 | 1 | pos | pos | pos | 2+ | - | 27/5 | - | - | 6.5 |
| MIC55 | 1 | pT1cpN0M0 | 2 | 1 | 2 | 3 | pos | neg | pos | neg | - | 17/0 | - | - | 1.8 |
| MIC57 | 2 | T1N1M0 | 2 | 2 | 2 | 2 | pos | pos | pos | neg | - | 14/1 | - | - | 1.3 |
| MIC59 | 2a | T2N0M0 | 2 | 1 | 3 | 2 | neg | pos | pos | neg | - | 21/0 | - | - | 3.0 |
| MIC60 | 1 | T1CN0M0 | 2 | 1 | 3 | 3 | neg | pos | pos | 3+ | - | 5/0 | - | - | 1.7 |
| MIC61 | 3b | T4bN1M0 | 1 | 1 | 2 | 1 | pos | neg | pos | 3+ | - | 4/1 | - | - | 2.5 |
| MIC63 | 3b | T4bN1M0 | 2 | 1 | 3 | 2 | pos | pos | pos | neg | - | 15/3 | - | - | 4.0 |
| MIC64 | 2 | T2N0i+M0 | 2 | 1 | 3 | 3 | neg | pos | pos | 3+ | - | 10/0 | - | - | 3.0 |
| MIC66 | 2 | T1CN0M0 | 2 | 1 | 3 | 2 | neg | neg | pos | 2+ | - | 13/0 | - | - | 2.0 |
| MIC68 | 3 | pT3N1M0 | 3 | 3 | 2 | 3 | pos | neg | neg | neg | - | 18/2 | pos | - | 8.0 |
| MIC81 | 2 | T1N2M0 | 1 | 1 | 2 | 1 | neg | pos | pos | 3+ | - | 25/8 | - | - | 1.2 |
| MIC82 | 3b | T4bN3M0 | 3 | 3 | 3 | 3 | pos | neg | neg | 2+ | - | 22/22 | - | - | 7.0 |
| MIC85 | 2a | T1cN2M0 | 2 | 1 | 3 | 2 | pos | pos | pos | neg | - | 17/5 | - | - | 2.0 |
| MIC88 | 2 | T2N1M0 | 3 | 3 | 3 | 3 | neg | pos | pos | 3+ | - | 37/2 | - | - | 1.5 |
| MIC9 | 3b | T4N3M0 | 1 | 1 | 2 | 1 | pos | neg | pos | 3+ | - | 22/20 | - | - | 2.0 |
| MIC93 | 3 | T2N3M0 | 2 | 1 | 3 | 3 | pos | pos | pos | 2+ | FISH neg | 31/23 | - | - | 4.0 |
| MIC96 | 2 | pT1cpN1M0 | 2 | 1 | 3 | 2 | neg | pos | pos | neg | - | 28/1 | - | - | 2.0 |
| MIC99 | 3 | T3N1M0 | 2 | 2 | 3 | 2 | pos | neg | pos | 3+ | - | 19/2 | - | - | 6.0 |
| SM29 | 1 | T2N0M0 | 3 | 3 | 3 | 3 | neg | neg | neg | neg | - | 4/0 | pos | pos | 2.0 |

**Abbreviations**: ^1^TNM, Tumor size (T), Lymph node status (N), and Metastasis presence (M); ^2^Scarff-Bloom Richardson graduate system modified by Elston and Ellis (histologic grade); ^3^Lymphovascular invasion; ^4^Progesterone receptor; ^5^Estrogen receptor; ^6^Immunohistochemistry assay; ^7^Fluorescence *in situ* hybridization; *Lymph node status (number of investigated lymph nodes/number of affected lymph nodes); ^#^sentinel lymph node.
