# Supplementary material for: Lymphovascular invasion and histologic grade are associated with specific genomic profiles in invasive carcinomas of the breast
Source: Tumour Biol. 2014 Nov 13;36(3):1835–48. doi: 10.1007/s13277-014-2786-z (PMC4375298; doi:10.1007/s13277-014-2786-z)
Supplement: Supplementary file 6 — (DOCX 22 kb) [file 13277_2014_2786_MOESM5_ESM.docx]

**Supplementary Table S2.** Array-CGH data summary of the 57 breast carcinomas: classification according to LVI status and histologic grade (SBR), total number of copy number aberrations, and number of each type of event (gain, loss, high copy gain and homozygous loss).

| **Sample ID** | **LVI** | **SBR^1^** | **Gain** | **Loss** | **High copy gain** | **Homozygous loss** | **Total CNA** |
| --- | --- | --- | --- | --- | --- | --- | --- |
| **MIC10** | Pos | 2 | 41 | 39 | 6 | 0 | 86 |
| **MIC120** | Pos | 3 | 27 | 12 | 0 | 0 | 39 |
| **MIC134** | neg | 3 | 23 | 5 | 1 | 0 | 29 |
| **MIC137** | neg | 3 | 30 | 16 | 0 | 0 | 46 |
| **MIC141** | pos | 3 | 35 | 18 | 0 | 1 | 54 |
| **MIC143** | neg | 3 | 33 | 25 | 2 | 0 | 60 |
| **MIC144** | pos | 3 | 38 | 18 | 0 | 1 | 57 |
| **MIC145** | neg | 3 | 39 | 34 | 8 | 1 | 82 |
| **MIC147** | pos | 3 | 11 | 8 | 2 | 0 | 21 |
| **MIC148** | neg | 3 | 29 | 26 | 3 | 0 | 58 |
| **MIC149** | pos | 3 | 38 | 17 | 9 | 0 | 64 |
| **MIC152** | pos | 3 | 7 | 0 | 1 | 0 | 8 |
| **MIC189** | neg | 3 | 50 | 40 | 0 | 0 | 90 |
| **MIC195** | neg | 1 | 19 | 0 | 2 | 0 | 21 |
| **MIC20** | pos | 3 | 57 | 54 | 3 | 0 | 114 |
| **MIC208** | pos | 3 | 15 | 20 | 3 | 0 | 38 |
| **MIC21** | neg | 3 | 1 | 1 | 0 | 0 | 2 |
| **MIC211** | pos | 3 | 18 | 15 | 3 | 0 | 36 |
| **MIC218** | neg | 3 | 29 | 67 | 0 | 0 | 96 |
| **MIC22** | neg | 1 | 22 | 18 | 0 | 0 | 40 |
| **MIC224** | neg | 3 | 48 | 36 | 1 | 2 | 87 |
| **MIC225** | neg | 2 | 32 | 22 | 4 | 0 | 58 |
| **MIC228** | neg | 3 | 39 | 28 | 4 | 0 | 71 |
| **MIC229** | neg | 3 | 0 | 1 | 0 | 0 | 1 |
| **MIC234** | neg | 3 | 50 | 51 | 0 | 0 | 101 |
| **MIC235** | neg | 3 | 31 | 33 | 5 | 0 | 69 |
| **MIC31** | neg | 2 | 89 | 74 | 28 | 6 | 197 |
| **MIC33** | pos | 1 | 7 | 6 | 5 | 0 | 18 |
| **MIC34** | neg | 2 | 29 | 14 | 0 | 0 | 43 |
| **MIC37** | neg | 1 | 19 | 30 | 0 | 0 | 49 |
| **MIC38** | neg | 1 | 3 | 2 | 0 | 0 | 5 |
| **MIC40** | neg | 3 | 11 | 9 | 0 | 0 | 20 |
| **MIC41** | pos | 3 | 50 | 53 | 2 | 0 | 105 |
| **MIC42** | neg | 2 | 12 | 0 | 0 | 0 | 12 |
| **MIC43** | pos | 3 | 68 | 48 | 3 | 0 | 119 |
| **MIC44** | neg | 2 | 17 | 11 | 0 | 0 | 28 |
| **MIC46** | neg | 3 | 45 | 28 | 4 | 0 | 77 |
| **MIC47** | pos | 3 | 25 | 9 | 4 | 0 | 38 |
| **MIC48** | pos | 1 | 7 | 9 | 0 | 0 | 16 |
| **MIC55** | pos | 2 | 10 | 14 | 0 | 0 | 24 |
| **MIC57** | pos | 2 | 0 | 3 | 0 | 0 | 3 |
| **MIC59** | neg | 2 | 12 | 1 | 0 | 0 | 13 |
| **MIC60** | neg | 2 | 9 | 7 | 4 | 0 | 20 |
| **MIC61** | pos | 1 | 36 | 73 | 0 | 0 | 109 |
| **MIC63** | pos | 2 | 27 | 5 | 1 | 0 | 33 |
| **MIC64** | neg | 2 | 8 | 9 | 0 | 0 | 17 |
| **MIC66** | neg | 2 | 7 | 12 | 0 | 0 | 19 |
| **MIC68** | pos | 3 | 41 | 47 | 0 | 0 | 88 |
| **MIC81** | neg | 1 | 12 | 17 | 0 | 0 | 29 |
| **MIC82** | pos | 3 | 101 | 89 | 8 | 2 | 200 |
| **MIC85** | pos | 2 | 1 | 0 | 0 | 0 | 1 |
| **MIC88** | neg | 3 | 0 | 0 | 0 | 0 | 0 |
| **MIC9** | pos | 1 | 3 | 0 | 0 | 0 | 3 |
| **MIC93** | pos | 2 | 10 | 1 | 0 | 0 | 11 |
| **MIC96** | neg | 2 | 8 | 29 | 0 | 0 | 37 |
| **MIC99** | pos | 2 | 3 | 0 | 1 | 0 | 4 |
| **SM29** | neg | 3 | 45 | 41 | 5 | 0 | 91 |

1. Scarff-Bloom-Richardson (SBR) modified by Elston and Ellis (Elston and Ellis, 1991). Somatically acquired copy number alterations (CNAs) were identified with the Nexus software 7.0 (Biodiscovery), using the FASST2 segmentation algorithm. We defined the following settings: a minimum of 5 consecutive affected probes (effective resolution of ~200 Kb for CNA calling), a significance threshold set at 1x10^-8^, and threshold log_2_ ratio Cy3/Cy5 of 0.3 and 1.4 for gain or high copy gain, respectively, and −0.3 and −1.1 for loss and homozygous loss, respectively.
