# Supplementary material for: Lymphovascular invasion and histologic grade are associated with specific genomic profiles in invasive carcinomas of the breast
Source: Tumour Biol. 2014 Nov 13;36(3):1835–48. doi: 10.1007/s13277-014-2786-z (PMC4375298; doi:10.1007/s13277-014-2786-z)
Supplement: Supplementary file 7 — (DOCX 17 kb) [file 13277_2014_2786_MOESM6_ESM.docx]

**Supplementary Table S4.** Chromosomal regions significantly associated with histologic grade III breast tumors (bold) compared to low-grade breast tumors (grades I + II). Genomic mapping, type of CNA, and genomic size are indicated as well as the difference in the frequency of the event in the two clinical categories (grade III and low-grade). Positive difference values (%) indicate rearrangements more frequently detected in the grade III group; conversely, negative difference values (%) indicate CNAs more frequently detected in the low-grade group.

| **Genomic mapping (Hg19)** | **Cytoband** | **Event type** | **Region length** | **Difference (%)** | **p-value** |
| --- | --- | --- | --- | --- | --- |
| **chr8:100,370,430-102,948,519** | **q22.2 - q22.3** | **CN Gain** | **2578089** | **35.6** | **0.007** |
| **chr8:102,986,143-104,515,702** | **q22.3** | **CN Gain** | **1529559** | **35.6** | **0.007** |
| **chr8:119,378,911-120,303,004** | **q24.12** | **CN Gain** | **924093** | **35.6** | **0.007** |
| **chr8:123,697,373-130,534,640** | **q24.13 - q24.21** | **CN Gain** | **6837267** | **35.6** | **0.007** |
| **chr20:4,501,033-4,687,670** | **p13** | **CN Gain** | **186637** | **29.0** | **0.002** |
| **chr4:44,365,354-45,636,982** | **p13 - p12** | **CN Loss** | **1271628** | **28.4** | **0.007** |
| **chr4:182,227,735-183,781,811** | **q34.3 - q35.1** | **CN Loss** | **1554076** | **28.4** | **0.007** |
| **chr9:23,365,289-23,517,075** | **p21.3** | **CN Loss** | **151786** | **28.4** | **0.007** |
| **chr9:26,150,711-26,557,216** | **p21.2** | **CN Loss** | **406505** | **28.4** | **0.007** |
| **chr5:16,484,385-16,605,527** | **p15.1** | **CN Gain** | **121142** | **28.4** | **0.007** |
| **chr7:632,740-1,628,634** | **p22.3** | **CN Gain** | **995894** | **28.4** | **0.007** |
| **chr7:4,331,952-5,256,244** | **p22.2 - p22.1** | **CN Gain** | **924292** | **28.4** | **0.007** |
| **chr5:56,409,216-59,147,681** | **q11.2 - q12.1** | **CN Loss** | **2738465** | **25.8** | **0.005** |
| **chr5:59,750,082-66,470,894** | **q12.1 - q12.3** | **CN Loss** | **6720812** | **25.8** | **0.005** |
| **chr5:70,748,823-74,061,021** | **q13.2 - q13.3** | **CN Loss** | **3312198** | **25.8** | **0.005** |
| **chr5:81,917,751-94,551,836** | **q14.2 - q15** | **CN Loss** | **12634085** | **25.8** | **0.005** |
| **chr5:98,038,385-111,282,729** | **q15 - q22.1** | **CN Loss** | **13244344** | **25.8** | **0.005** |
| **chr5:113,249,132-118,122,138** | **q22.3 - q23.1** | **CN Loss** | **4873006** | **25.8** | **0.005** |
| **chr9:26,725,105-30,864,850** | **p21.2 - p21.1** | **CN Loss** | **4139745** | **25.8** | **0.005** |
| **chr12:15,989,092-17,373,378** | **p12.3** | **CN Loss** | **1384286** | **25.8** | **0.005** |
| **chr21:22,512,995-25,866,788** | **q21.1 - q21.2** | **CN Loss** | **3353793** | **25.8** | **0.005** |
| **chr10:0-1,099,007** | **p15.3** | **CN Gain** | **1099007** | **25.8** | **0.005** |
| **chr10:28,691,475-31,124,806** | **p12.1 - p11.23** | **CN Gain** | **2433331** | **25.8** | **0.005** |
| **chr19:29,180,564-30,617,071** | **q12** | **CN Gain** | **1436507** | **25.8** | **0.005** |
| chr16:16,236,300-22,999,655 | p13.11 - p12.2 | CN Gain | 6763355 | -35.8 | 0.005 |
| chr16:16,236,300-22,999,655 | p13.11 - p12.2 | CN Gain | 6763355 | -35.8 | 0.005 |
| chr16:7,553,076-8,001,201 | p13.3 - p13.2 | CN Gain | 448125 | -33.0 | 0.003 |
| chr16:7,553,076-8,001,201 | p13.3 - p13.2 | CN Gain | 448125 | -33.0 | 0.003 |
| chr16:12,983,721-14,505,236 | p13.12 | CN Gain | 1521515 | -32.6 | 0.005 |
| chr16:25,212,918-27,094,899 | p12.1 | CN Gain | 1881981 | -32.6 | 0.005 |
| chr16:5,502,628-7,273,687 | p13.3 | CN Gain | 1771059 | -32.0 | 0.007 |
| chr16:68,937,994-69,135,316 | q22.1 | CN Loss | 197322 | -26.9 | 0.002 |
| chr16:69,569,397-69,952,036 | q22.1 | CN Loss | 382639 | -26.9 | 0.002 |

Comparison using the STAC analysis (*p* ≤ 0.01; differential threshold of 25%); genomic segments with >75% of CNV overlap (according to DGV data) were excluded.
