# Supplementary material for: Lymphovascular invasion and histologic grade are associated with specific genomic profiles in invasive carcinomas of the breast
Source: Tumour Biol. 2014 Nov 13;36(3):1835–48. doi: 10.1007/s13277-014-2786-z (PMC4375298; doi:10.1007/s13277-014-2786-z)
Supplement: Supplementary file 8 — (DOCX 14 kb) [file 13277_2014_2786_MOESM7_ESM.docx]

**Supplementary Table S5.** Chromosomal regions significantly associated with LVI-positive breast tumors (bold) compared to LVI-negative breast tumors. Genomic mapping, type of CNA, and genomic size are indicated as well as the difference in the frequency of the event in the two clinical categories (LVI-positive and LVI-negative). Positive difference values (%) indicate rearrangements more frequently detected in the LVI-positive group; conversely, negative difference values (%) indicate CNAs more frequently detected in the LVI-negative group.

| Genomic mapping (Hg19) | Cytoband | | Event type | Region Length | Difference (%) | p-value |
| --- | --- | --- | --- | --- | --- | --- |
| chr11:123,655,355-123,721,051 | | **q24.1** | **CN Loss** | **65696** | **25.7** | **0.01** |
| chr11:128,855,246-128,915,412 | | **q24.3** | **CN Loss** | **60166** | **26.6** | **0.02** |
| chr16:80,711,108-80,819,896 | | q23.2 | CN Loss | 108788 | -26.3 | 0.02 |
| chr16:75,680,945-76,278,203 | | q23.1 | CN Loss | 597258 | -25.5 | 0.03 |
| chr16:4,828,405-5,502,628 | | p13.3 | CN Gain | 674223 | -25.5 | 0.03 |
| chr16:8,465,780-10,584,125 | | p13.2 - p13.13 | CN Gain | 2118345 | -25.5 | 0.03 |
| chr16:12,253,740-15,137,951 | | p13.13 - p13.11 | CN Gain | 2884211 | -25.5 | 0.03 |
| chr16:15,796,712-20,911,903 | | p13.11 - p12.3 | CN Gain | 5115191 | -25.5 | 0.03 |
| chr16:23,892,453-27,593,308 | | p12.2 - p12.1 | CN Gain | 3700855 | -25.5 | 0.03 |

Comparison using the STAC analysis (*p* ≤ 0.01; differential threshold of 25%); genomic segments with >75% of CNV overlap (according to DGV data) were excluded.
