# Supplementary material for: Lymphovascular invasion and histologic grade are associated with specific genomic profiles in invasive carcinomas of the breast
Source: Tumour Biol. 2014 Nov 13;36(3):1835–48. doi: 10.1007/s13277-014-2786-z (PMC4375298; doi:10.1007/s13277-014-2786-z)
Supplement: Supplementary file 9 — (DOCX 36 kb) [file 13277_2014_2786_MOESM8_ESM.docx]

**Supplementary Table S6.** List of differentially expressed genes in histologic grade III breast tumors compared to grades I+II tumors (fold-change |2|; p=0.001).

| Gene Symbol | Cytoband | Fold Change | p-value |  | Gene Symbol | Cytoband | Fold Change | p-value |
| --- | --- | --- | --- | --- | --- | --- | --- | --- |
| *S100A8* | 1q21.3 | 22.0278 | 0.001 |  | *GRP* | 18q21.32 | -8.9074 | 0.001 |
| *DEFB1* | 8p23.1 | 14.2762 | 0.001 |  | *CYP4X1* | 1p33 | -8.1935 | 0.001 |
| *ADM* | 11p15.4 | 7.0605 | 0.001 |  | *SUSD3* | 9q22.31 | -8.0443 | 0.001 |
| *MMP1* | 11q22.2 | 6.7464 | 0.001 |  | *ABAT* | 16p13.2 | -7.3311 | 0.001 |
| *MCM10* | 10p13 | 6.4085 | 0.001 |  | *EVL* | 14q32.2 | -6.9978 | 0.001 |
| *TMSB15A* | Xq22.1 | 6.2266 | 0.001 |  | *KLHDC9* | 1q23.3 | -6.9593 | 0.001 |
| *SOX11* | 2p25.2 | 5.5269 | 0.001 |  | *C6orf211* | 6q25.1 | -6.9305 | 0.001 |
| *YBX2* | 17p13.1 | 5.3408 | 0.001 |  | *NEFH* | 22q12.2 | -6.3975 | 0.001 |
| *KRT81* | 12q13.13 | 5.3214 | 0.001 |  | *CX3CR1* | 3p22.2 | -5.3859 | 0.001 |
| *E2F8* | 11p15.1 | 5.2362 | 0.001 |  | *SMOC2* | 6q27 | -5.3324 | 0.001 |
| *NANOS1* | 10q26.11 | 5.1092 | 0.001 |  | *BTG2* | 1q32.1 | -5.3206 | 0.001 |
| *CDC45L* | 22q11.21 | 5.0596 | 0.001 |  | *FAM198B* | 4q32.1 | -5.0859 | 0.001 |
| *CDCA7* | 2q31.1 | 4.6827 | 0.001 |  | *CCDC74B* | 2q21.1 | -5.0592 | 0.001 |
| *CDCA5* | 11q13.1 | 4.6527 | 0.001 |  | *SH3BGRL* | Xq21.1 | -4.9315 | 0.001 |
| *CTSL2* | 9q22.33 | 4.6284 | 0.001 |  | *ZG16B* | 16p13.3 | -4.7622 | 0.001 |
| *ASPM* | 1q31.3 | 4.6081 | 0.001 |  | *RERG* | 12p12.3 | -4.4616 | 0.001 |
| *C15orf42* | 15q26.1 | 4.4926 | 0.001 |  | *SPARC* | 5q33.1 | -4.3650 | 0.001 |
| *LAD1* | 1q32.1 | 4.4734 | 0.001 |  | *C5orf4* | 5q33.2 | -4.3529 | 0.001 |
| *FAM64A* | 17p13.2 | 4.4701 | 0.001 |  | *FBN1* | 15q21.1 | -4.1728 | 0.001 |
| *CCNB2* | 15q22.2 | 4.4450 | 0.001 |  | *DKK3* | 11p15.3 | -4.0412 | 0.001 |
| *CDCA2* | 8p21.2 | 4.4282 | 0.001 |  | *KCNK15* | 20q13.12 | -4.0190 | 0.001 |
| *DSCC1* | 8q24.12 | 4.2221 | 0.001 |  | *IGF2* | 11p15.5 | -4.0068 | 0.001 |
| *TROAP* | 12q13.12 | 4.1656 | 0.001 |  | *LOC100286909* | 1q32.2 | -3.9859 | 0.001 |
| *C1orf135* | 1p36.11 | 4.0552 | 0.001 |  | *RBMS3* | 3p24.1 | -3.9709 | 0.001 |
| *ASPM* | 1q31.3 | 4.0242 | 0.001 |  | *INPP4B* | 4q31.21 | -3.9629 | 0.001 |
| *OSBPL3* | 7p15.3 | 4.0145 | 0.001 |  | *IL6ST* | 5q11.2 | -3.8891 | 0.001 |
| *SKA3* | 13q12.11 | 3.8765 | 0.001 |  | *HTRA1* | 10q26.13 | -3.8640 | 0.001 |
| *LOC441795* | 17q21.31 | 3.8622 | 0.001 |  | *LOXL1* | 15q24.1 | -3.8450 | 0.001 |
| *VEGFA* | 6p21.1 | 3.8391 | 0.001 |  | *C20orf103* | 20p12.2 | -3.8168 | 0.001 |
| *NCAPH* | 2q11.2 | 3.8180 | 0.001 |  | *COL5A2* | 2q32.2 | -3.6982 | 0.001 |
| *PIF1* | 15q22.31 | 3.7523 | 0.001 |  | *CSNK1G3* | 5q23.2 | -3.5421 | 0.001 |
| *UHRF1* | 19p13.3 | 3.7263 | 0.001 |  | *MKL2* | 16p13.12 | -3.4677 | 0.001 |
| *STMN1* | 1p36.11 | 3.6697 | 0.001 |  | *DCN* | 12q21.33 | -3.4581 | 0.001 |
| *RAD51* | 15q15.1 | 3.6466 | 0.001 |  | *ANKRA2* | 5q13.2 | -3.4530 | 0.001 |
| *CENPN* | 16q23.2 | 3.5498 | 0.001 |  | *ARMCX2* | Xq22.1 | -3.3996 | 0.001 |
| *GSG2* | 17p13.3 | 3.5117 | 0.001 |  | *SULF2* | 20q13.12 | -3.3774 | 0.001 |
| *POLQ* | 3q13.33 | 3.4903 | 0.001 |  | *PAPD4* | 5q14.1 | -3.3686 | 0.001 |
| *E2F1* | 20q11.22 | 3.4629 | 0.001 |  | *KCTD6* | 3p14.3 | -3.3580 | 0.001 |
| *LOC100287566* | 16q21 | 3.4407 | 0.001 |  | *CTSK* | 1q21.2 | -3.3292 | 0.001 |
| *AMD1* | 6q21 | 3.3778 | 0.001 |  | *AKAP9* | 7q21.2 | -3.2745 | 0.001 |
| *NCAPG* | 4p15.32 | 3.3657 | 0.001 |  | *RSPH1* | 21q22.3 | -3.2612 | 0.001 |
| *SGOL1* | 3p24.3 | 3.3317 | 0.001 |  | *TRIM45* | 1p13.1 | -3.2391 | 0.001 |
| *PSAT1* | 9q21.2 | 3.3010 | 0.001 |  | *THBS2* | 6q27 | -3.2245 | 0.001 |
| *CDC2* | 10q21.2 | 3.2947 | 0.001 |  | *PBX1* | 1q23.3 | -3.2209 | 0.001 |
| *CIT* | 12q24.23 | 3.2651 | 0.001 |  | *ITPR1* | 3p26.2 | -3.1949 | 0.001 |
| *DLAT* | 11q23.1 | 3.2596 | 0.001 |  | *EPHX2* | 8p21.1 | -3.1389 | 0.001 |
| *RAD54L* | 1p33 | 3.2283 | 0.001 |  | *SLC44A1* | 9q31.1 | -3.1296 | 0.001 |
| *YBX1* | 1p34.2 | 3.0915 | 0.001 |  | *KIF16B* | 20p12.1 | -3.1266 | 0.001 |
| *CDCA8* | 1p34.3 | 3.0766 | 0.001 |  | *FANK1* | 10q26.2 | -3.1013 | 0.001 |
| *EIF2C2* | 8q24.3 | 3.0430 | 0.001 |  | *TMEM25* | 11q23.3 | -3.0735 | 0.001 |
| *DEPDC1* | 1p31.2 | 3.0272 | 0.001 |  | *KLHDC2* | 14q22.1 | -3.0466 | 0.001 |
| *FOXK2* | 17q25.3 | 3.0078 | 0.001 |  | *COL3A1* | 2q32.2 | -3.0096 | 0.001 |
| *OIP5* | 15q15.1 | 3.0057 | 0.001 |  | *BMI1* | 10p12.31 | -3.0010 | 0.001 |
| *KPNA2* | 17q24.2 | 2.9747 | 0.001 |  | *KIAA1370* | 15q21.2 | -2.9594 | 0.001 |
| *RIOK3* | 18q11.2 | 2.9398 | 0.001 |  | *DOCK10* | 2q36.2 | -2.9323 | 0.001 |
| *MEX3A* | 1q22 | 2.9036 | 0.001 |  | *ADD3* | 10q25.2 | -2.9265 | 0.001 |
| *PFKP* | 10p15.2 | 2.8824 | 0.001 |  | *APBB2* | 4p14 | -2.8588 | 0.001 |
| *GPSM2* | 1p13.3 | 2.8536 | 0.001 |  | *C5orf41* | 5q35.2 | -2.8480 | 0.001 |
| *PRC1* | 15q26.1 | 2.8516 | 0.001 |  | *ELP2* | 18q12.2 | -2.7849 | 0.001 |
| *PTTG2* | 4p14 | 2.8274 | 0.001 |  | *DOCK1* | 10q26.2 | -2.7644 | 0.001 |
| *SNX5* | 20p11.23 | 2.8093 | 0.001 |  | *RUNX1* | 21q22.12 | -2.7046 | 0.001 |
| *DHCR7* | 11q13.4 | 2.7701 | 0.001 |  | *COL1A1* | 17q21.33 | -2.6943 | 0.001 |
| *LMNB2* | 19p13.3 | 2.7667 | 0.001 |  | *FAM63A* | 1q21.2 | -2.6481 | 0.001 |
| *MUC4* | 3q29 | 2.7598 | 0.001 |  | *PTPLAD1* | 15q22.31 | -2.5989 | 0.001 |
| *CDT1* | 16q24.3 | 2.7573 | 0.001 |  | *EXOC6* | 10q23.33 | -2.5973 | 0.001 |
| *C7orf68* | 7q32.1 | 2.7347 | 0.001 |  | *ZBED5* | 11p15.3 | -2.5755 | 0.001 |
| *RACGAP1* | 12q13.13 | 2.7306 | 0.001 |  | *TAF7* | 5q31.3 | -2.5500 | 0.001 |
| *PABPC1* | 8q22.3 | 2.6862 | 0.001 |  | *DMXL1* | 5q23.1 | -2.5424 | 0.001 |
| *PKMYT1* | 16p13.3 | 2.6239 | 0.001 |  | *FAM174A* | 5q21.1 | -2.5293 | 0.001 |
| *OR7E156P* | 13q21.31 | 2.6120 | 0.001 |  | *C5orf44* | 5q12.3 | -2.4906 | 0.001 |
| *CTPS* | 1p34.2 | 2.5904 | 0.001 |  | *DNAJC1* | 10p12.31 | -2.4838 | 0.001 |
| *OR7E24* | 19p13.2 | 2.5851 | 0.001 |  | *JKAMP* | 14q23.1 | -2.4610 | 0.001 |
| *BID* | 22q11.21 | 2.5795 | 0.001 |  | *TGOLN2* | 2p11.2 | -2.4517 | 0.001 |
| *TK1* | 17q25.3 | 2.4969 | 0.001 |  | *C16orf72* | 16p13.2 | -2.4469 | 0.001 |
| *DLGAP5* | 14q22.3 | 2.4907 | 0.001 |  | *KIFAP3* | 1q24.2 | -2.4412 | 0.001 |
| *CASC5* | 15q15.1 | 2.4394 | 0.001 |  | *IQCK* | 16p12.3 | -2.4386 | 0.001 |
| *SRCRB4D* | 7q11.23 | 2.4127 | 0.001 |  | *RALGAPA1* | 14q13.2 | -2.4321 | 0.001 |
| *TIMM8A* | Xq22.1 | 2.3526 | 0.001 |  | *CRY2* | 11p11.2 | -2.4234 | 0.001 |
| *PPAT* | 4q12 | 2.3280 | 0.001 |  | *HEYL* | 1p34.2 | -2.4162 | 0.001 |
| *PATL1* | 11q12.1 | 2.3224 | 0.001 |  | *CCNDBP1* | 15q15.2 | -2.3542 | 0.001 |
| *KIF23* | 15q23 | 2.2857 | 0.001 |  | *DOK1* | 2p13.1 | -2.3367 | 0.001 |
| *FANCI* | 15q26.1 | 2.2734 | 0.001 |  | *KTN1* | 14q22.3 | -2.3254 | 0.001 |
| *ITPR3* | 6p21.31 | 2.2524 | 0.001 |  | *C5orf15* | 5q31.1 | -2.3210 | 0.001 |
| *NCBP2* | 3q29 | 2.2359 | 0.001 |  | *PDGFRB* | 5q33.1 | -2.2959 | 0.001 |
| *SMC4* | 3q26.1 | 2.2358 | 0.001 |  | *LHFP* | 13q13.3 | -2.2797 | 0.001 |
| *HDAC2* | 6q22.1 | 2.2086 | 0.001 |  | *TMEM9B* | 11p15.4 | -2.2683 | 0.001 |
| *CCT5* | 5p15.2 | 2.1852 | 0.001 |  | *AP3B1* | 5q14.1 | -2.2584 | 0.001 |
| *NFIL3* | 9q22.31 | 2.1819 | 0.001 |  | *PSPC1* | 13q12.11 | -2.2278 | 0.001 |
| *HMMR* | 5q34 | 2.1764 | 0.001 |  | *PPOX* | 1q23.3 | -2.2213 | 0.001 |
| *TCF3* | 19p13.3 | 2.1486 | 0.001 |  | *EFHA1* | 13q12.11 | -2.1919 | 0.001 |
| *KPNA4* | 3q26.1 | 2.1209 | 0.001 |  | *C11orf17* | 11p15.4 | -2.1728 | 0.001 |
| *PSMG1* | 21q22.2 | 2.1110 | 0.001 |  | *ZC3H6* | 2q13 | -2.1503 | 0.001 |
| *DHFR* | 5q14.1 | 2.0910 | 0.001 |  | *P4HTM* | 3p21.31 | -2.1341 | 0.001 |
| *SMOX* | 20p13 | 2.0594 | 0.001 |  | *QSOX1* | 1q25.2 | -2.1171 | 0.001 |
| *SNX3* | 6q21 | 2.0136 | 0.001 |  | *C5orf45* | 5q35.3 | -2.0972 | 0.001 |
| *EZH2* | 7q36.1 | 2.0062 | 0.001 |  | *COMMD3* | 10p12.31 | -2.0721 | 0.001 |
|  |  |  |  |  | *ZBTB20* | 3q13.31 | -2.0306 | 0.001 |
|  |  |  |  |  | *C16orf52* | 16p12.1 | -2.0299 | 0.001 |
|  |  |  |  |  | *GLTSCR2* | 19q13.32 | -2.0245 | 0.001 |
|  |  |  |  |  | *NOTCH2* | 1p12 | -2.0224 | 0.001 |
|  |  |  |  |  | *SKP1* | 5q31.1 | -2.0034 | 0.001 |
