# Supplementary material for: Lymphovascular invasion and histologic grade are associated with specific genomic profiles in invasive carcinomas of the breast
Source: Tumour Biol. 2014 Nov 13;36(3):1835–48. doi: 10.1007/s13277-014-2786-z (PMC4375298; doi:10.1007/s13277-014-2786-z)
Supplement: Supplementary file 10 — (DOCX 21 kb) [file 13277_2014_2786_MOESM9_ESM.docx]

**Supplementary Table S7.** List of differentially expressed genes in lymphovascular invasion positive tumors compared to negative tumors (fold-change |2|; p<0.05).

| Gene Symbol | Cytoband | Fold-change | p-value |  | Gene Symbol | Cytoband | Fold-change | p-value |
| --- | --- | --- | --- | --- | --- | --- | --- | --- |
| *C19orf33* | 19q13.2 | 4.4608 | 0.0200 |  | *GFRA1* | 10q25.3 | -4.4772 | 0.0210 |
| *LGALS7B* | 19q13.2 | 3.5713 | 0.0070 |  | *MFI2* | 3q29 | -4.0174 | 0.0290 |
| *CPE* | 4q32.3 | 3.0436 | 0.0110 |  | *TBX21* | 17q21.32 | -3.8633 | 0.0376 |
| *AGBL2* | 11p11.2 | 2.9353 | 0.0280 |  | *KRT15* | 17q21.2 | -3.6634 | 0.0160 |
| *ARSG* | 17q24.2 | 2.7475 | 0.0440 |  | *NFASC* | 1q32.1 | -3.3951 | 0.0220 |
| *UMOD* | 16p12.3 | 2.6808 | 0.0250 |  | *FZD5* | 2q33.3 | -3.1834 | 0.0080 |
| *C13orf31* | 13q14.11 | 2.6631 | 0.0259 |  | *TCF7L1* | 2p11.2 | -3.0653 | 0.0470 |
| *MYCBPAP* | 17q21.33 | 2.5776 | 0.0320 |  | *MYBPC2* | 19q13.33 | -2.8301 | 0.0210 |
| *CXXC4* | 4q24 | 2.5767 | 0.0440 |  | *CRABP1* | 15q25.1 | -2.7727 | 0.0170 |
| *MED1* | 17q12 | 2.5187 | 0.0330 |  | *CIB2* | 15q25.1 | -2.5553 | 0.0180 |
| *SHISA5* | 3p21.31 | 2.5084 | 0.0020 |  | *DUSP3* | 17q21.31 | -2.5390 | 0.0010 |
| *SRGAP1* | 12q14.2 | 2.4905 | 0.0070 |  | *ALKBH5* | 17p11.2 | -2.4245 | 0.0210 |
| *GALNT10* | 5q33.2 | 2.4694 | 0.0020 |  | *IGFBP5* | 2q35 | -2.4043 | 0.0330 |
| *RADIL* | 7p22.1 | 2.4537 | 0.0310 |  | *HS3ST1* | 4p15.33 | -2.3679 | 0.0100 |
| *EIF2S3* | Xp22.11 | 2.4163 | 0.0020 |  | *DTYMK* | 2q37.3 | -2.3431 | 0.0060 |
| *AKAP8L* | 19p13.12 | 2.3939 | 0.0040 |  | *PRR4* | 12p13.2 | -2.3235 | 0.0050 |
| *COL17A1* | 10q25.1 | 2.3719 | 0.0290 |  | *BMP7* | 20q13.31 | -2.3214 | 0.0260 |
| *ZNF727* | 7q11.21 | 2.3136 | 0.0020 |  | *CCDC85C* | 14q32.2 | -2.2768 | 0.0050 |
| *NETO2* | 16q12.1 | 2.2703 | 0.0470 |  | *OTOF* | 2p23.3 | -2.2415 | 0.0210 |
| *LFNG* | 7p22.2 | 2.2557 | 0.0350 |  | *RTN4R* | 22q11.21 | -2.2186 | 0.0070 |
| *ZNF714* | 19p12 | 2.2527 | 0.0310 |  | *NGEF* | 2q37.1 | -2.2076 | 0.0460 |
| *HELZ* | 17q24.2 | 2.2127 | 0.0070 |  | *FREQ* | 9q34.11 | -2.2036 | 0.0110 |
| *CTH* | 1p31.1 | 2.1794 | 0.0030 |  | *HSPC159* | 2p14 | -2.1074 | 0.0310 |
| *MARVELD3* | 16q22.3 | 2.1583 | 0.0120 |  | *NCOA7* | 6q22.32 | -2.1022 | 0.0320 |
| *MT1G* | 16q13 | 2.1265 | 0.0220 |  | *LOC441245* | 7q11.21 | -2.0910 | 0.0150 |
| *C7orf46* | 7p15.3 | 2.1198 | 0.0250 |  | *BEGAIN* | 14q32.2 | -2.0586 | 0.0370 |
| *SNHG5* | 6q14.3 | 2.1177 | 0.0170 |  | *TARDBP* | 1p36.22 | -2.0536 | 0.0060 |
| *KCNJ14* | 19q13.32 | 2.0974 | 0.0060 |  | *CBR1* | 21q22.12 | -2.0439 | 0.0210 |
| *MTAP* | 9p21.3 | 2.0802 | 0.0220 |  | *ZSCAN2* | 15q25.2 | -2.0230 | 0.0250 |
| *LOC651250* | 17q24.2 | 2.0727 | 0.0170 |  | *NCF2* | 1q25.3 | -2.0202 | 0.0400 |
| *MT1E* | 16q13 | 2.0693 | 0.0320 |  | *FGF7* | 15q21.1 | -2.0100 | 0.0330 |
| *DPY19L1P1* | 7p14.3 | 2.0661 | 0.0140 |  |  |  |  |  |
| *HNRNPA3* | 2q31.2 | 2.0632 | 0.0010 |  |  |  |  |  |
| *FOXP2* | 7q31.1 | 2.0620 | 0.0280 |  |  |  |  |  |
| *ZNF671* | 19q13.43 | 2.0613 | 0.0450 |  |  |  |  |  |
| *C1orf63* | 1p36.11 | 2.0421 | 0.0260 |  |  |  |  |  |
| *ZNF234* | 19q13.31 | 2.0377 | 0.0100 |  |  |  |  |  |
| *FOXP1* | 3p14.1 | 2.0353 | 0.0170 |  |  |  |  |  |
| *GUSBL2* | 6p22.1 | 2.0038 | 0.0320 |  |  |  |  |  |
